# CCL18 signaling from tumor-associated macrophages activates fibroblasts to adopt a chemoresistance-inducing phenotype

**DOI:** 10.1038/s41388-022-02540-2

**Published:** 2022-11-22

**Authors:** Wenfeng Zeng, Lixiong Xiong, Wei Wu, Shunrong Li, Jiang Liu, Linbing Yang, Liyan Lao, Penghan Huang, Mengmeng Zhang, Huiping Chen, Nanyan Miao, Zhirong Lin, Zifei Liu, Xinyu Yang, Jiayi Wang, Pei Wang, Erwei Song, Yandan Yao, Yan Nie, Jianing Chen, Di Huang

**Affiliations:** 1 Guangdong Provincial Key Laboratory of Malignant Tumor Epigenetics and Gene Regulation, Medical Research Center, Sun Yat-Sen Memorial Hospital, Sun Yat-Sen University, Guangzhou, 510120 China; 2 Breast Tumor Center, Sun Yat-Sen Memorial Hospital, Sun Yat-Sen University, Guangzhou, 510120 China; 3 Bioland Laboratory, Guangzhou, 510005 China; 4 Department of Pharmacy, the Sixth Affiliated Hospital, Sun Yat-Sen University, Guangzhou, 510655 China; 5 Department of Breast Surgery, Peking University People’s Hospital, Peking University, Beijing, 100044 China; 6 Shenshan Medical Center, Sun Yat-sen Memorial Hospital, Sun Yat-sen University, Shanwei, 516621 China

**Keywords:** Cell signalling, Breast cancer, Cancer microenvironment, Cancer stem cells

## Abstract

The heterogeneity of cancer-associated fibroblasts (CAFs) might be ascribed to differences in origin. CD10 and GPR77 have been reported to identify a chemoresistance-inducing CAF subset in breast cancer. However, the precise mechanism for the formation of the CD10^+^GPR77^+^ CAFs remains unknown. In this study, we found that CCL18 expression was positively correlated with the density of CD10^+^GPR77^+^ CAFs in breast cancer and associated with a poor response to chemotherapy. Moreover, CCL18 secreted by tumor-associated macrophages (TAMs) activated a CD10^+^GPR77^+^ CAF phenotype in normal breast-resident fibroblasts (NBFs), which could then enrich cancer stem cells (CSCs) and induce chemoresistance in breast cancer cells. Mechanistically, CCL18 activated NF-κB signaling via PITPNM3 and thus enhanced the production of IL-6 and IL-8. Furthermore, intratumoral CCL18 injection significantly induced the activation of NBFs and the chemoresistance of xenografts in vivo. In addition, targeting CCL18 by anti-CCL18 antibody could inhibit the formation of CD10^+^GPR77^+^ CAFs and recover the chemosensitivity in vivo, leading to effective tumor control. Collectively, these findings reveal that inflammatory signaling crosstalk between TAMs and fibroblasts is responsible for the formation of the CD10^+^GPR77^+^ CAFs, suggesting CCL18–PITPNM3 signaling is a potential therapeutic target to block the activation of this specific CAF subtype and tumor chemoresistance.

## Introduction

Cancer-associated fibroblasts are abundant in the stroma of a variety of malignant tumors, have been proven to generally promote tumor progression by inducing angiogenesis [1], promoting tumor proliferation [2], conferring therapeutic resistance [3] and so on. However, nonselective elimination of CAFs was found to result in disease exacerbation in clinic [4] and in mouse pancreatic cancer model [5, 6], suggesting that the functional heterogeneity of fibroblasts in tumor microenvironment is really significant. Recently, emerging evidence has helped identify specific CAF subsets related to various tumor characteristics, including cancer formation [3, 7], chemoresistance [3] and immunosuppression [8, 9]. Therefore, it’s necessary to identify the functional CAF subsets by specific surface markers and uncover the underlying activation mechanism, which could pave the path to developing a precise anticancer therapy targeting the specific CAF subset.

CAF heterogeneity might be ascribed to the different potential cellular sources of CAFs and variant activation mechanisms, which remain unknown. CAFs are potentially derived from several cell types that are recruited to the tumor or undergo differentiation in situ, including normal resident fibroblasts and stellate cells [10–12], bone marrow mesenchymal stem cells (BMSCs) [10, 13], pericytes [11, 14], adipocytes [15], epithelial cells and endothelial cells that undergo epithelial to mesenchymal transition (EMT) [10, 16] or endothelial–mesenchymal transition (EndMT) [10, 17]. In general, activation of local tissue-resident fibroblasts and stellate cells is considered the major pathway of CAF generation [18, 19]. Fibroblasts are quiescent in normal tissues and can be activated during wound healing. The long-held notion that tumors are ‘wounds that do not heal’ implies the inappropriate activation of fibroblasts [20]. In malignant tumors, tumor cells can activate the inflammatory pathway in normal fibroblasts by secreting cytokines, such as TGF-β1 [21], PDGF [22], and IL-1β [23], resulting in the transformation into CAFs. Furthermore, exosomes released by tumor cells deliver growth factors, cytokines, functional DNA fragments and coding and non-coding RNAs to fibroblasts to induce their activation and differentiation [24, 25]. In addition, the stromal cells in tumor microenvironment can also activate the normal fibroblasts. M2 polarized TAMs [26] or tumor microenvironment stress [27] can induce normal fibroblasts upregulating α-SMA expression, leading to the differentiation to CAFs. However, the precise mechanism by which specific subtypes of CAFs are activated is still unclear.

A unique protumorigenic CAF subset expressing CD10 and GPR77 provides a survival niche for cancer stem cells (CSCs) during chemotherapy for breast and lung cancer [3] and was associated with drug resistance in polyploid giant cancer cells [28]. These CAFs are driven by persistent NF-κB activation via GPR77 signaling-induced p65 phosphorylation and acetylation. However, the initiation factors responsible for NF-κB activation have yet to be clarified. In this study, we aimed to explore the origins of the CD10^+^GPR77^+^ CAF subset and the underlying mechanism, with the goal of finding strategies to inhibit the enrichment of this subset and suppress the protumorigenic effects in early-stage malignancies.

## Results

### Breast cancers with different chemotherapeutic responses exhibit conspicuously distinct cytokine profiles in their tumor microenvironment

**Fig. 1:**
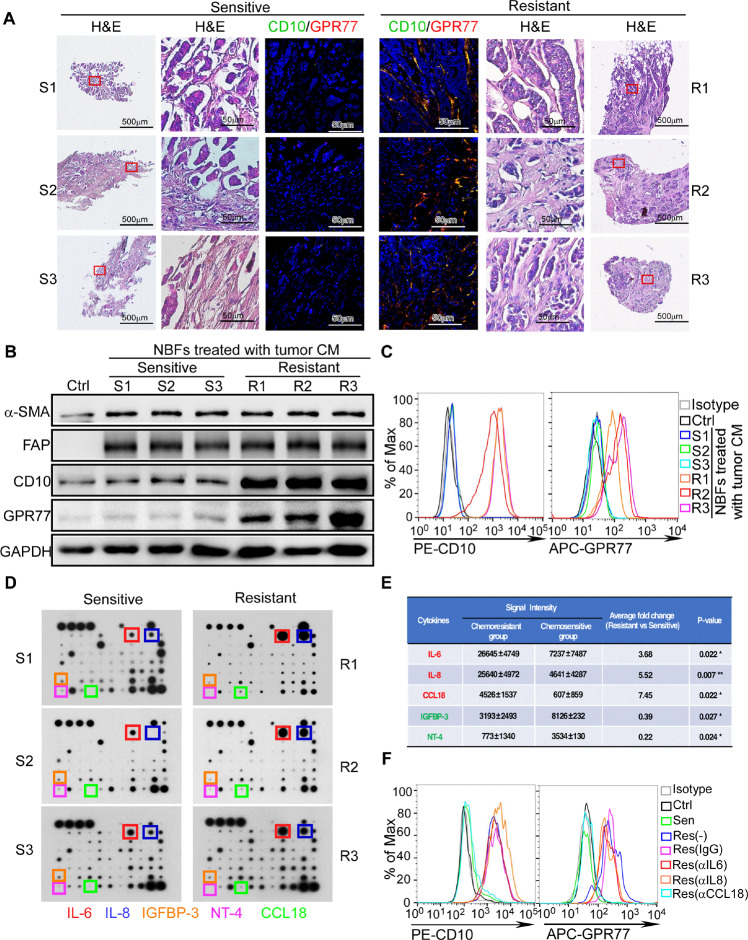
Breast cancers with different chemotherapeutic responses exhibit conspicuously distinct cytokine profiles in their tumor microenvironment. **A** Representative images of H&E staining and CD10/GPR77 immunofluorescent staining in serial sections of the pre-treatment breast cancer biopsies of chemosensitive (*n* = 3) and chemoresistant (*n* = 3) patients. **B**, **C** Western blotting (**B**) for α-SMA, FAP, CD10, and GPR77 and flow cytometric analysis (**C**) for CD10 and GPR77 in primary normal breast fibroblasts (NBFs) treated with pre-treatment tumor conditional medium (CM). **D** Cytokine arrays of pre-treatment tumor CM, squares indicate the cytokines with significant changes. **E** Signal intensity of indicated cytokines in the cytokine arrays and their relative fold change between the chemoresistant (*n* = 3) and chemosensitive tumors (*n* = 3) **F** Flow cytometric analysis for CD10 and GPR77 in NBFs treated with chemosensitive or chemoresistant tumor CM added without or with neutralizing antibodies against IL6(αIL6), IL8(αIL8) or CCL18(αCCL18). The patients with complete remission (CR) or partial remission (PR) were classified as chemosensitive, while those with stable disease (SD) or progressive disease (PD) were chemoresistant.

To investigate whether the chemoresistance-inducing CD10^+^GPR77^+^ CAF subset was derived from normal breast tissues and activated or differentiated by the tumor microenvironment in situ, we isolated different cell types from normal breast tissues, including NBFs, pericytes, adipocytes, epithelial cells and endothelial cells, and mesenchymal stem cells (MSCs) from bone marrow, followed by treatment with the conditioned medium (CM) of fresh breast cancer tissues. These fresh breast cancer tissues were obtained from vacuum-assisted biopsies prior to chemotherapy and cultured for CM collection. Then, the patients received neoadjuvant chemotherapy and were divided into the chemosensitive or chemoresistant group according to treatment response. Consistent with the previous reports [3], the chemoresistant patients (those with progressive disease (PD) or stable disease (SD)) had more CD10^+^GPR77^+^ CAFs in their biopsy samples than the chemosensitive patients (those with a complete response (CR) or partial response (PR)) (Fig. 1A and Supplementary Fig. 1A). Interestingly, chemoresistant and chemosensitive tumor CM could not upregulate CD10 and GPR77 expression in MSCs, pericytes, adipocytes, epithelial cells and endothelial cells (Supplementary Fig.1B), while CD10 and GPR77 were abundantly expressed in NBFs treated with chemoresistant tumor CM (Supplementary Fig.1B), which was validated by Western blotting (Fig. 1B and Supplementary Fig. 1C), flow cytometric analysis (Fig. 1C and Supplementary Fig. 1D) and real-time quantitative PCR (RT-qPCR) (Supplementary Fig. 1E). Moreover, we observed that NBFs treated with tumor CM exhibit CAF-like phenotype, as shown by the elevated expression of the classical myofibroblast markers α-smooth muscle actin (α-SMA) and fibroblast activation protein (FAP), as well as the increased production of extracellular matrix (ECM), collagen type I alpha 1 chain (COL1A1) and collagen type III alpha 1 chain (COL3A1) (Fig. 1B and Supplementary Fig. 1C, E). Since CD10^+^GPR77^+^ CAFs induce chemoresistance by secreting IL-6 and IL-8 [3], we examined the production of these cytokines by RT-qPCR in NBFs treated with CM from different tumors and found that chemoresistant tumor CM significantly induced the production of IL-6 and IL-8 (Supplementary Fig. 1E). These data suggested that the CD10^+^GPR77^+^ CAF subset was derived from NBFs and might be activated by cytokines in the breast cancer microenvironment.

To identify the cytokines responsible for activating chemoresistance-inducing fibroblasts, the cytokine profiles of tumor CM from chemosensitive breast cancer with rare CD10^+^GPR77^+^ CAFs and from chemoresistant breast cancer with abundant CD10^+^GPR77^+^ CAFs were analyzed using a RayBio Human Cytokine Antibody Array (Fig. 1D). The gray intensity analysis showed that three cytokines, IL-6, IL-8, and CCL18, were abundant in the chemoresistant tumor samples with plentiful CD10^+^GPR77^+^ CAFs (Fig. 1E), which were validated by ELISA (Supplementary Fig. 1F). We then added neutralizing antibodies against CCL18, IL-6 and IL-8 to NBFs treated with fresh chemoresistant tumor CM. Interestingly, we found that blocking CCL18, but not IL-6 and IL-8, significantly inhibited the upregulation of CD10 and GPR77 in NBFs exposed to chemoresistant tumor CM (Fig. 1F and Supplementary Fig. 1G, H). These data suggested that CCL18 from tumor CM might mediate the activation of chemoresistance-inducing fibroblasts.

### The intratumoral accumulation of CCL18^+^ tumor-associated macrophages is associated with the abundance of CD10^+^GPR77^+^ CAFs and chemoresistance

**Fig. 2:**
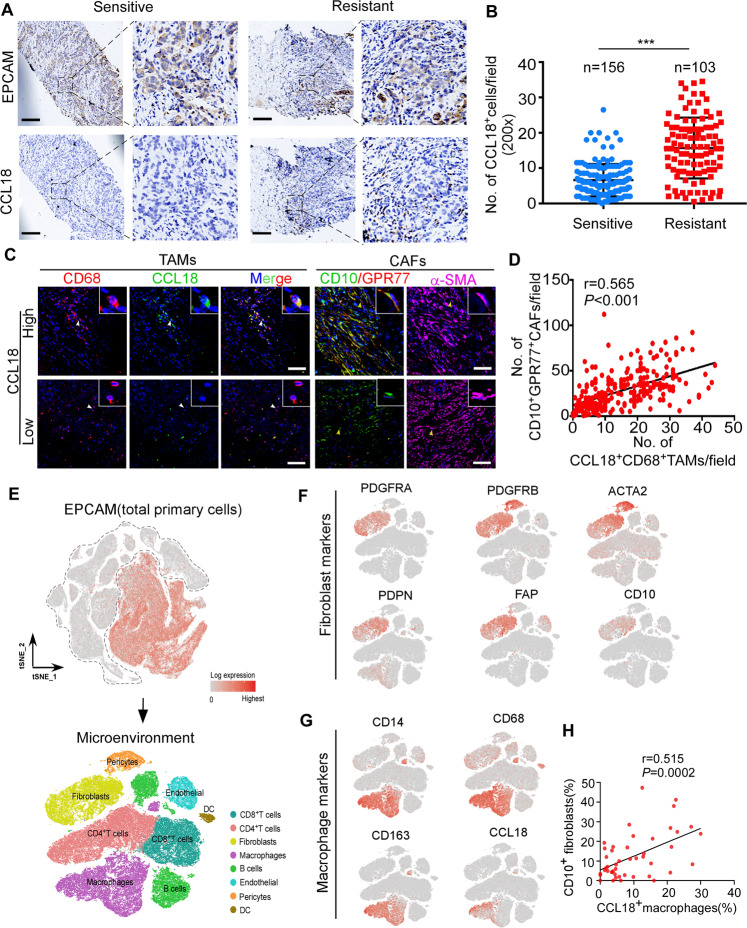
The intratumoral accumulation of CCL18^+^ tumor-associated macrophages is associated with the abundance of CD10^+^GPR77^+^ CAFs and chemoresistance. **A** Representative images of immunohistochemical staining for CCL18 and EPCAM in pre-treatment breast cancer biopsies (*n* = 259, *n* = 156 in chemosensitive group and *n* = 103 in chemoresistant group). Scale bars, 500 μm. **B** The counts of CCL18^+^ cells in pre-treatment breast cancer biopsies with different chemotherapeutic responses. Mean ± SEM, ***P < 0.001 by two-tailed Student’s *t* test (each dot represents the mean of the counts of at least five random fields from each tumor section). **C** Representative images of immunofluorescent staining of CD68, CCL18, CD10, GPR77 and α-SMA in serial sections of breast cancer samples with high or low CCL18 expression. Scale bars, 50 μm. The arrowheads denote the area of higher-magnification images shown in the top-right corner. **D** The correlation between the number of CD10^+^GPR77^+^ CAFs and CCL18^+^ TAMs in breast cancer samples. The Pearson’s correlation coefficient *r* value and *P* values were determined by two-tailed Pearson correlation coefficient test (*n* = 259). **E** (Upper) EPCAM expression on t-SNE map of combined scRNA-seq transcriptomes of total breast tissue cells from GEO: GSE161529 and Pan-cancer TME Blueprint, and (lower) the EPCAM-negative cells were reclustered to eight microenvironment populations according to their representative markers. **F**, **G** Expression plots of (**F**) fibroblast markers (PDGFRA, PDGFRB, ACTA2, PDPN, FAP and CD10) and (**G**) macrophage markers (CD14, CD68, CD163, and CCL18) were exhibited on t-SNE layout. **H** The correlation between the percentage of CD10 positive cells in fibroblasts with the percentage of CCL18 positive cells in macrophages in breast cancer samples based on scRNA-seq transcriptomes. The Pearson’s correlation coefficient *r* value and *P* values were determined by two-tailed Pearson correlation coefficient test (*n* = 46).

To further validate the cytokine array results, we performed immunohistochemistry staining for CCL18 in breast tumor biopsies from 259 breast cancer patients before neoadjuvant chemotherapy and correlated CCL18^+^ cell count with chemotherapeutic response. We found that the CCL18^+^ cell density in the tumor samples from chemoresistant patients (*n* = 103) was dramatically higher than that from responsive patients (*n* = 156) (Fig. 2A, B), implying that CCL18 participates in breast cancer chemoresistance. However, we treated breast cancer cells MCF-7 with recombinant CCL18 for 2 weeks, and then challenged the tumor cells with docetaxel or cisplatin (Supplementary Fig. 2A). Unexpectedly, CCL18 treatment didn’t enhance the survival of breast tumor cells under chemotherapy (Supplementary Fig. 2A). We then investigated whether CCL18 was responsible for the activation of chemoresistance-inducing CAFs with CD10 and GPR77 expression. Based on the BioGPS dataset (http://biogps.org/#goto=genereport&id=6362), CCL18 is a chemokine predominantly produced by monocyte-derived cells with M2 phenotype and TAMs [29]. We performed immunofluorescent staining of CCL18 and macrophage marker CD68 (Fig. 2C), as well as CCL18 and M2 macrophage marker CD163 (Supplementary Fig. 2B), respectively. In addition, CD10^+^GPR77^+^ CAFs were identified by triple immunostaining for CD10, GPR77 and -SMA in the serial sections. We found that the infiltration of CCL18^+^ TAMs was positively correlated with the number of CD10^+^GPR77^+^ CAFs (Fig. 2C, D and Supplementary Fig. 2B, C; CCL18^+^CD68^+^ cells: Pearson’s *r* = 0.565, *P* < 0.001, *n* = 259; CCL18^+^CD163^+^ cells: Pearson’s *r* = 0.45, *P* < 0.001, *n* = 259). Furthermore, we analyzed this correlation by chemosensitivity stratification. Although the infiltration of CCL18^+^TAMs was much less in the chemosensitive cohort than chemoresistant cohort (Fig. 2B), the positive correlation between CD10^+^GPR77^+^ CAFs and CCL18^+^ TAMs was found across both chemoresistant and chemosensitive breast cancer cohorts (Supplementary Fig. 2D). Morever, there was a positive correlation between CCL18^+^ TAMs and CD10^+^GPR77^+^ CAFs in different molecular subtypes of tumors, including 178 cases of hormone receptor positive (HR^+^) and human epidermal growth factor receptor-2 amplification (HER2^+^), 46 cases of HR^-^HER2^+^ and 35 cases of triple-negative breast cancer (TNBC) (Supplementary Fig. 2E).

To further evaluate the correlation between CCL18^+^ TAMs and CD10^+^GPR77^+^ CAFs, we analyzed single-cell RNA sequencing (scRNA-seq) data for 46 cases of treatment-naive breast cancer from GEO series GSE161529 and 14 cases of treatment-naive breast cancer from the pancancer TME blueprint (https://lambrechtslab.sites.vib.be/en/pan-cancer-blueprint-tumour-microenvironment-0). After quality filtering by read count and a low number of mitochondrial reads, 195,905 total cells were retained for subsequent analysis. Using the Seurat analysis package, data from individual samples were integrated and clustered based on canonical correlation analysis (CCA), and the combined profiles were visualized by t-distributed stochastic neighbor embedding (t-SNE) dimension reduction. Epithelial cells were first annotated based on the expression of the canonical marker EPCAM (Fig. 2E). To further probe the identity of cells within the ductal microenvironment, the EPCAM^+^ epithelial cell clusters were removed, and the remaining cells were reclustered to yield ten nonepithelial clusters (Fig. 2E). Then, these cell clusters were annotated as eight microenvironment populations (Fig. 2E) based on the expression of marker genes such as PDGFRA, PDGFRB, ACTA2, PDPN and FAP for fibroblasts (Fig. 2F) and CD14, CD163 and CD68 for monocytes/macrophages (Fig. 2G). We found that 89.9% of CCL18^+^ cells were CD68^+^ macrophages and 88.4% of CCL18^+^ cells were CD163^+^ macrophages (Fig. 2G). In addition, patients were grouped according to CCL18 expression in TAMs (Supplementary Fig. 2F). Notably, the patients with a high frequency of CCL18^+^ macrophages have higher expression of the M2 markers (MRC1, VCAN, CD163 and TGFB1) in comparison to those with low CCL18^+^ macrophage enrichment (Supplementary Fig. 2F, G), suggesting that CCL18 was robustly expressed by TAMs with M2 phenotype rather than by other cell types. Furthermore, we explored the correlation between CCL18^+^ TAM infiltration and CD10^+^GPR77^+^ CAFs in each sample. However, scRNA-seq is an inherently “low depth” analysis, as current methods can capture only a small fraction of the ~300 K transcripts in individual cells [30]. In these scRNA-seq data, we found that GPR77 expression was too low for analysis. CD10 was highly expressed in fibroblasts (Fig. 2F), and the percentage of CD10^+^ CAFs was positively associated with the infiltration of CCL18^+^ TAMs (Fig. 2H). We further analyzed clinically stratified breast cancer cases, including 8 cases of TNBC, 6 cases with HR^-^HER2^+^, and 18 cases of HR^+^, and found that all subtypes of breast tumors showed a trend of a positive correlation between CD10^+^ CAFs and CCL18^+^ TAMs, but the *P* values did not indicate statistical significance due to the limited number of cases (Supplementary Fig. 2H). These data suggested that the intratumoral accumulation of CCL18^+^ TAMs is associated with an abundance of chemoresistance-inducing CD10^+^GPR77^+^ CAFs.

### CCL18 produced by TAMs mediates the chemoresistance-inducing phenotype polarization in NBFs

**Fig. 3:**
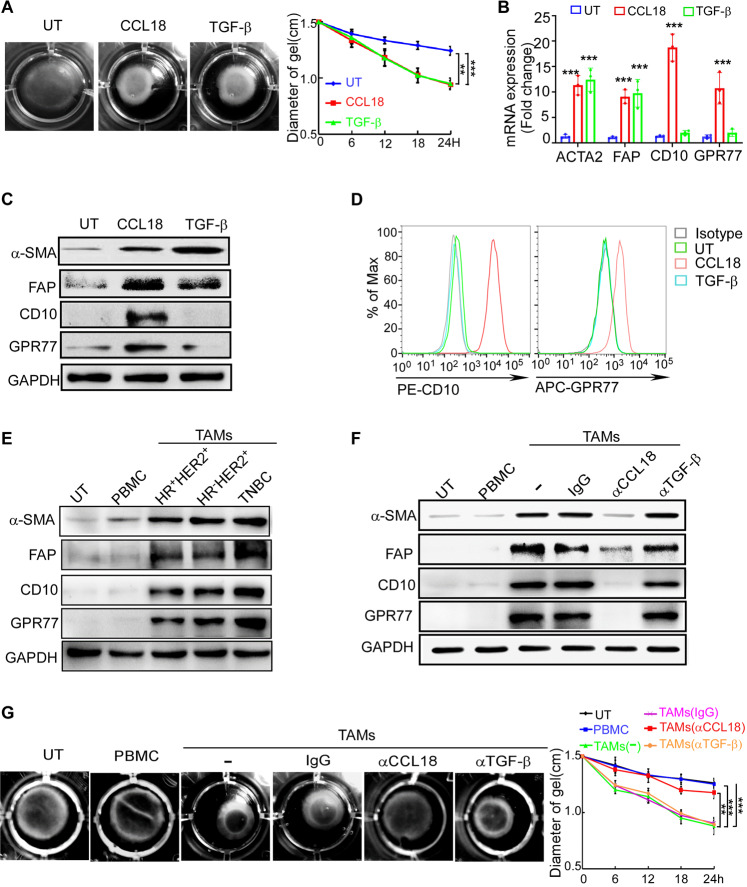
CCL18 produced by TAMs mediates the chemoresistance-inducing phenotype polarization in NBFs. **A** Representative images(left) and diameters(right) of collagen gel contraction assay for NBFs with or without CCL18 and TGF-β treatment was determined by three-dimensional collagen matrices (*n* = 3). **B**, **C** QRT–PCR(**B**) and western blotting(**C**) for α-SMA, FAP, CD10 and GPR77 expression in NBFs with or without CCL18 and TGF-β treatment (*n* = 3). **D** Representative flow cytometric analysis for CD10 and GPR77 in NBFs with indicated treatment (*n* = 3). **E** Representative western blotting for α-SMA, FAP, CD10, and GPR77 in NBFs cultured alone (UT) or co-cultured with PBMC or TAMs isolated from different subtypes of breast cancer (*n* = 3). **F** Western blotting for α-SMA, FAP, CD10 and GPR77 in NBFs cultured alone (UT) or co-cultured with PBMC or TAMs pretreated with control IgG or neutralizing antibodies against CCL18(αCCL18) or TGF-β(αTGF-β) (*n* = 3). **G** Representative images(left) and diameters(right) of collagen gel contraction assay for NBFs treated as in **F** (*n* = 3). Data expressed as mean ± SEM, **P < 0.01, ***P < 0.001 compared with untreated NBFs (**A**, **B**) or NBFs co-cultured with untreated TAMs (**G**) by two-tailed one-way ANOVA with Dunnett’s multiple comparison test.

To investigate whether CCL18 mediates the activation of fibroblasts in the tumor stroma, NBFs isolated from reduction mammaplasty samples were treated with recombinant CCL18 and TGF-β, which is typically used as a positive control for fibroblast activation [31, 32]. CAFs are considered as highly activated fibroblasts [33] and have strong ECM remodeling ability [34], which can be detected by a widely-used and classical functional assay, collagen gel contraction assay [35]. We found that CCL18 treatment enhanced fibroblast-mediated collagen contraction to an equivalent extent as TGF-β (Fig. 3A). In addition, both CCL18 and TGF-β induced the upregulation of α-SMA and FAP (Fig. 3B, C and Supplementary Fig. 3A–C), but only CCL18 upregulated CD10 and GPR77 (Fig. 3B–D and Supplementary Fig. 3A, D). To further investigate the contribution of TAM-derived CCL18 in the activation of chemoresistance-inducing fibroblasts, primary TAMs were isolated from breast cancer samples of different molecular subtypes and then cocultured with NBFs. We found that TAMs from all subtypes activated a chemoresistance-inducing phenotype in NBFs (Fig. 3E and Supplementary Fig. 3E). Moreover, TAM-induced fibroblast activation was markedly inhibited by the neutralizing antibody to CCL18, not TGF-β (Fig. 3F, G and Supplementary Fig. 3F), suggesting that TAMs activated a chemoresistance-inducing phenotype in NBFs via CCL18, not TGF-β.

### NBFs activated by CCL18-producing TAMs mediate chemoresistance and CSC enrichment by secreting IL-6 and IL-8

**Fig. 4:**
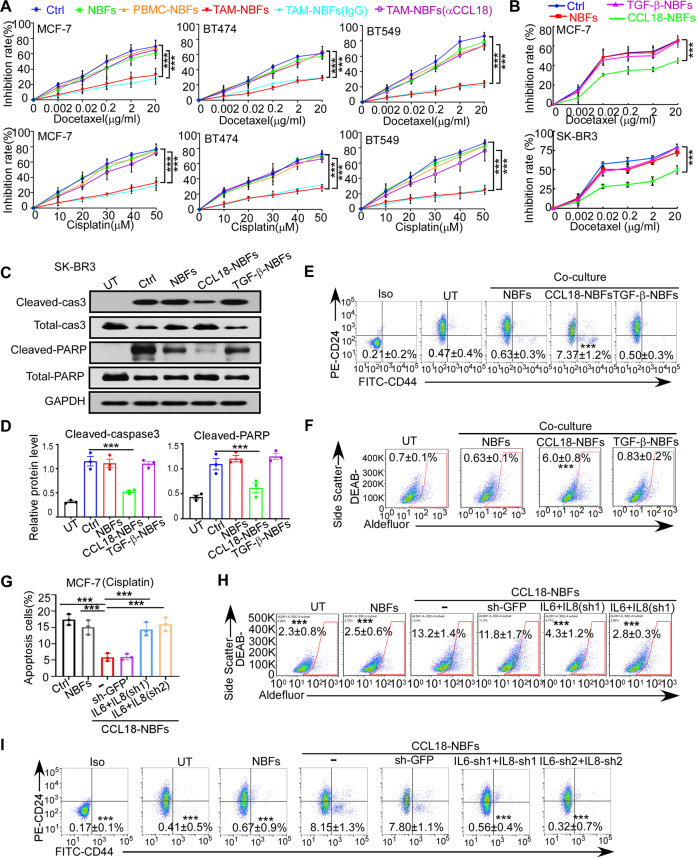
NBFs activated by CCL18-producing TAMs mediate chemoresistance and CSC enrichment by secreting IL-6 and IL-8. **A** The growth inhibition rate of docetaxel (up) and cisplatin (down) on MCF-7, BT474 or BT549 cultured alone (Ctrl) or co-cultured with NBFs underwent indicated treatment (*n* = 3). **B** The growth inhibition rate of docetaxel on MCF-7 (up) and SK-BR3 (down) cells cultured alone (Ctrl) or co-cultured with NBFs underwent indicated treatment (*n* = 3). **C**, **D** Representative western blotting (**C**) and quantification (**D**) for cleavage of caspase-3 and PARP in SK-BR3 cells cultured alone (Ctrl) or co-cultured with NBFs underwent indicated treatment and challenged with cisplatin (*n* = 3). **E**, **F** The percentage of CD44^+^CD24^-^ (**E**) and ALDH1^+^(**F**) cells in MCF-7 cells cultured alone (UT) or co-cultured with NBFs underwent indicated treatment. **G**–**I** The apoptosis after cisplatin treatment (**G**), the ALDH1^+^ (**H**) and the CD44^+^CD24^-^(**I**) proportion of MCF-7 cells cultured alone or co-cultured with untreated or CCL18-treated NBFs transduced with GFP shRNA or IL-6 and IL-8 shRNA (*n* = 3). Data expressed as mean ± SEM, ***P < 0.001 compared with NBFs co-cultured with untreated TAMs (**A**), untreated NBFs (**B**, **D**–**F**) or CCL18-treated NBFs without shRNA transduction (**G**–**I**) by two-tailed one-way ANOVA with Dunnett’s multiple comparison test.

To further investigate the role of TAM-activated fibroblasts, we treated NBFs with CM from freshly isolated TAMs from breast cancers of different molecular subtypes. Then, the activated NBFs were cocultured with breast cancer cell lines of the same molecular subtype, such as HR^+^ MCF-7 cells, HER2^+^ BT474 cells and TNBC BT549 cells. Afterward, the cancer cells were retrieved from the cocultures and challenged with chemotherapeutics. We found that NBFs activated by TAMs from all subtypes enhanced the survival and reduced the apoptosis of cocultured breast cancer cells exposed to the chemotherapeutics docetaxel and cisplatin (Fig. 4A and Supplementary Fig. 4A). Interestingly, these TAM-induced effects were dramatically inhibited by the CCL18 neutralizing antibody (Fig. 4A and Supplementary Fig. 4A). To further investigate whether fibroblasts activated by different cytokines are heterogeneous, we cocultured NBFs activated by different cytokines with MCF-7 and SKBR3 breast cancer cells and then challenged the tumor cells with docetaxel or cisplatin. Interestingly, CCL18-activated NBFs, but not TGF-β-treated NBFs, enhanced the survival of cocultured tumor cells exposed to the chemotherapeutic docetaxel or cisplatin (Fig. 4B and Supplementary Fig. 4B). Consistently, compared with TGF-β-activated NBFs, CCL18-activated NBFs effectively reduced the chemotherapy-induced apoptosis of breast cancer cells (Fig. 4C, D and Supplementary Fig. 4C).

The existence of CSCs is significantly correlated with chemoresistance [36, 37]. We therefore evaluated the stemness characteristics of breast cancer cells cocultured with NBFs pretreated with various cytokines. In agreement with previous data, the proportions of CD44^+^CD24^-^ and ALDH1^+^ breast CSCs were dramatically increased among breast cancer cells cocultured with CCL18-activated NBFs but not with treatment-naive or TGF-β-treated NBFs (Fig. 4E, F). In addition, MCF-7 breast cancer cells cocultured with CCL18-activated NBFs generated significantly more mammospheres (Supplementary Fig. 4D). The above data suggested that the diversity of factors that initiate cell activation may be responsible for the heterogeneity of CAFs in the tumor microenvironment.

Since CCL18-activated NBFs have a phenotype and function similar to CD10^+^GPR77^+^ CAFs, we wondered whether CCL18-activated NBFs induce CSC enrichment and chemoresistance via IL-6 and IL-8, which are produced by CD10^+^GPR77^+^ CAFs [3]. We found that CCL18 but not TGF-β dramatically increased the production and secretion of IL-6 and IL-8 by NBFs, as determined by RT–qPCR (Supplementary Fig. 4E) and ELISA (Supplementary Fig. 4F). Next, we suppressed IL-6 and IL-8 production in CCL18-activated NBFs using shRNAs targeting IL-6 and IL-8 and verified the knockdown efficiency by ELISA (Supplementary Fig. 4G). Then we cocultured these NBFs with MCF-7 breast cancer cells, which were subsequently treated with chemotherapy. Interestingly, the introduction of IL-6 and IL-8 shRNAs into CCL18-activated NBFs significantly increased the chemotherapy-induced apoptosis of cocultured tumor cells (Fig. 4G and Supplementary Fig. 4H). Moreover, CSC enrichment among MCF-7 cells cocultured with CCL18-activated NBFs with IL-6 and IL-8 knockdown was dramatically reduced, compared with MCF-7 cells cocultured with CCL18-activated NBFs, which was reduced to nearly the level observed among cells without coculture (Fig. 4H, I and Supplementary Fig. 4I). Collectively, these data suggested that CCL18-activated NBFs enrich CSCs and induce chemoresistance by secreting IL-6 and IL-8.

### PITPNM3 mediates CCL18-induced fibroblast activation via NF-κB signaling

**Fig. 5:**
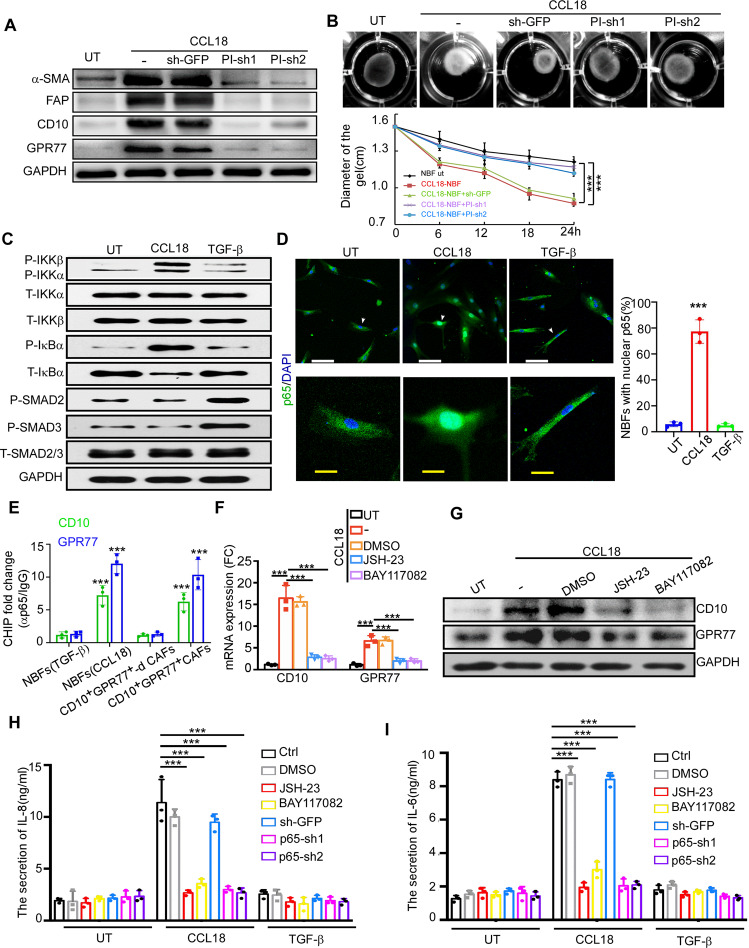
PITPNM3 mediates CCL18-induced fibroblast activation via NF-κB signaling. **A** Representative western blotting for α-SMA, FAP, CD10 and GPR77 in untreated (UT) or CCL18-treated NBFs transduced with shRNA against GFP or PITPNM3 (*n* = 3). **B** Representative images(up) and diameters(down) of collagen gel contraction assay for NBFs with indicated treatment (*n* = 3). **C** Representative western blotting for total expression and phosphorylation of IKK, IκB and SMAD2/3 in NBFs with indicated treatment (*n* = 3). **D** (left)Representative images of p65 immunofluorescent staining and (right) quantification of p65 nuclear translocation in NBFs with indicated treatment (*n* = 3). Scale bars (white line), 50 μm; Scale bars (yellow line), 5 μm. **E** Localizations of p65 to the promoters of CD10 and GPR77 genes in indicated fibroblasts were analyzed by ChIP assay using anti-p65 Ab or control IgG. **F**, **G** QRT–PCR (**F**) and representative western blotting (**G**) for CD10 and GPR77 expression in untreated (UT) or CCL18-activated NBFs treated with DMSO, JSH-23 or BAY117082 (*n* = 3). **H**, **I** ELISA for IL-8 (**H**) and IL-6 (**I**) levels in the conditioned medium of untreated, CCL18 or TGF-β treated NBFs in the presence of JSH-23, BAY117082 or transduced with GFP or p65 shRNAs (*n* = 3). JSH-23, an NF-κB inhibitor. BAY117082, an IKK inhibitor. Data expressed as mean ± SEM, ***P < 0.001 compared with NBFs co-cultured with untreated CCL18-activated NBFs (**B**, **F**, **H**, **I**) or TGF-β treated NBFs (**E**) by two-tailed one-way ANOVA with Dunnett’s multiple comparison test.

It has been reported that PITPNM3, CCR6 and CCR8 are putative receptors of CCL18 [38, 39]. PITPNM3 has been proven to be a functional CCL18 receptor in breast tumor cells [29] and T lymphocytes [40], while CCR6 and CCR8 have been proven to be expressed in T lymphocytes [39, 41]. To investigate the CCL18 receptor in fibroblasts, we performed flow cytometric analysis to determine the expression of PITPNM3, CCR6, and CCR8 in fibroblasts, with MDA-MB-231 cells or T lymphocytes as the positive control. We observed little CCR6 and CCR8 expression in fibroblasts, but the levels of PITPNM3 in both NBFs and CAFs were comparable to those in MDA-MB-231 cells (Supplementary Fig. 5A, B). Consistently, in the scRNA-seq analysis of the breast cancer microenvironment, CCR6 and CCR8 were predominantly expressed in lymphocytes, not fibroblasts (Supplementary Fig. 5C). As PITPNM3 expression was not evaluable in the scRNA-seq dataset due to excessive dropouts, we examined the expression of this protein by Western blotting and confirmed expression in NBFs (Supplementary Fig. 5D, E). These data implied that PITPNM3 may be a functional receptor for CCL18 in NBFs. Thus, we silenced PITPNM3 in NBFs using shRNA and found that PITPNM3 knockdown in NBFs markedly impeded the CCL18-induced upregulation of α-SMA, FAP, CD10 and GPR77 (Fig. 5A and Supplementary Fig. 5F, G). Moreover, collagen contraction by CCL18-activated NBFs was attenuated by PITPNM3 knockdown (Fig. 5B), suggesting that CCL18 induces NBF activation via PITPNM3.

The NF-κB signaling pathway has been confirmed to be essential for the epithelial–mesenchymal transition (EMT) of cancer cells induced by CCL18-producing TAMs [42]. In addition, IL-6 and IL-8 are recognized target genes of NF-κB-dependent transcription [43, 44]. Thus, we examined the activation of NF-κB signaling in NBFs treated with CCL18 and TGF-β. We found that CCL18 but not TGF-β dramatically increased the phosphorylation of IκB kinase (IKK) and IκB, resulting in IκB degradation (Fig. 5C and Supplementary Fig. 5H). However, TGF-β significantly induced the phosphorylation of SMAD2 and SMAD3 (Fig. 5C and Supplementary Fig. 5H), which was consistent with a previous report related to TGF-β-induced fibrosis [45]. Consistently, CCL18 but not TGF-β induced p65 nuclear translocation in NBFs (Fig. 5D), with a subsequent increase in NF-κB transcriptional activity (Supplementary Fig. 5I). Furthermore, we performed chromatin immunoprecipitation (ChIP) with an anti-p65 antibody to detect binding sites upstream of the CD10 and GPR77 expression cassettes. We found enhanced NF-κB binding to the CD10 and GPR77 promoters in CCL18-activated NBFs, which was equivalent to the positive control CD10^+^GPR77^+^ CAFs, but not in TGF-β-activated NBFs or non-CD10^+^GPR77^+^ CAFs (Fig. 5E), suggesting that CD10 and GPR77 are NF-κB target genes. Then, we utilized the pharmacologic inhibitor of NF-κB nuclear translocation 4-methyl-N1-(3-phenyl-propyl)-benzene-1,2-diamine (JSH-23), the IKK inhibitor BAY-117082 and two shRNAs targeting p65 to suppress NF-κB signaling. Strikingly, inhibition of NF-κB markedly decreased CD10 and GPR77 expression (Fig. 5F, G and Supplementary Fig. 5J) and IL-6 and IL-8 production (Fig. 5H, I) in CCL18-activated NBFs. Collectively, these data revealed that NF-κB is essential for CCL18-mediated fibroblast activation to the CD10^+^GPR77^+^ phenotype.

### CCL18 promotes breast cancer tumorigenesis and chemoresistance in vivo by activating NBFs

**Fig. 6:**
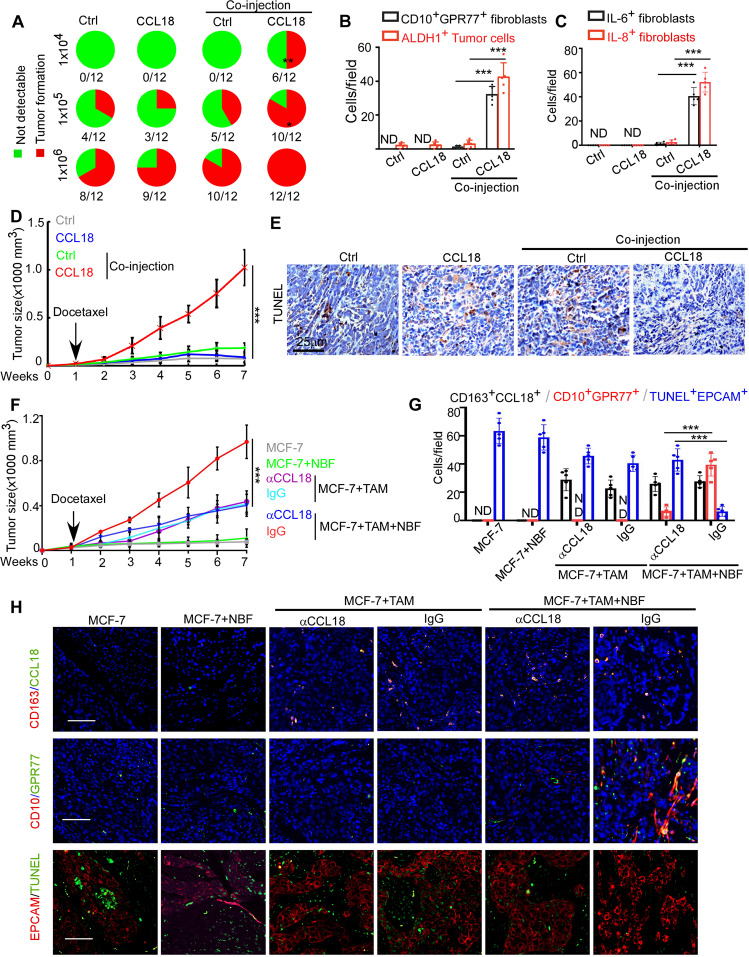
CCL18 promotes breast cancer tumorigenesis and chemoresistance in vivo by activating NBFs. **A** MCF-7 cells were implanted alone or co-injected with NBFs into the mammary fat pads of NOD/SCID mice, and recombinant CCL18 was administrated twice a week and the tumor formation were monitored for up to three months. Xenograft formation rates were shown (*n* = 12 per group). Green part represented the proportion of mice fail to develop tumors and red part represented the proportion of mice that developed tumors successfully. *P < 0.05, **P < 0.01 by Fisher’s exact test. **B** Quantification for CD10^+^GPR77^+^ fibroblasts(black) and ALDH1^+^ tumor cells(red) by immunofluorescent staining in the harvested tumors in **A** (*n* = 5 per group). **C** Quantification for the density of IL6^+^fibroblasts(black) and IL8^+^fibroblasts(red) determined by immunofluorescent staining in the harvested tumors in **A** (*n* = 5 per group). **D**, **E** MCF-7 cells were implanted alone or co-injected with NBFs and docetaxel was administrated one week after implantation concomitantly with or without recombinant CCL18. **D** Tumor growth was monitored for 7 weeks (mean ± SEM, *n* = 8 per group). **E** Representative images for TUNEL^+^ cells in the xenografts related to **D** (mean ± SEM, *n* = 8 per group). Scale bars, 25 μm. **F-H**, MCF-7 cells were implanted without or with NBFs and TAMs into the mammary fat pads of NOD/SCID mice and neutralizing antibody against CCL18 was used to block CCL18 signaling. Docetaxel was administrated 1 week after implantation. **F** Tumor growth was monitored for 7 weeks (mean ± SEM, *n* = 8 per group). **G** Quantification (*n* = 5 per group) and (**H**) representative immunofluorescent images for CD163^+^CCL18^+^ macrophages, CD10^+^GPR77^+^ fibroblasts and TUNEL^+^EPCAM^+^ tumor cells in the harvested tumors in **F**. Scale bars in **H** 50 μm. Data expressed as Mean ± SEM, ***P < 0.001 compared with xenografts formed by untreated MCF-7(**A**, **D**, **E**), xenografts formed by MCF-7 cells co-implanted with NBFs without CCL18 treatment (**B**, **C**) or xenografts formed by MCF-7 cells co-implanted with NBFs and TAMs without CCL18 neutralizing antibody treatment (**F**, **G**) by two-tailed one-way ANOVA with Dunnett’s multiple comparison test.

To examine the effect of CCL18 on NBF activation and function in vivo, we inoculated MCF-7 cells with or without NBFs into the mammary fat pads of NOD/SCID mice to establish a xenograft mouse model. These mice subsequently received intratumoral injections of recombinant CCL18 at a dosage of 0.1 mg/kg biweekly for 10 consecutive weeks. We found that CCL18 administration dramatically enhanced the tumorigenicity of MCF-7 cells serially transplanted with NBFs but had no effect on that of breast cancer xenografts without fibroblasts (Fig. 6A). In parallel, immunostaining showed that the proportion of ALDH1^+^ breast cancer cells was considerably higher in the xenografts formed by MCF-7 cells coinjected with NBFs and treated with CCL18 (Fig. 6B and Supplementary Fig. 6A), and the mice harboring these tumors also had a greater abundance of CD10^+^GRP77^+^ fibroblasts (Fig. 6B and Supplementary Fig. 6A). Consistently, the intratumoral injection of CCL18 into xenografts formed by MCF-7 cells co-implanted with NBFs promoted IL-6 and IL-8 secretion by fibroblasts, as determined by triple immunofluorescence staining for α-SMA, IL-6 and IL-8 (Fig. 6C and Supplementary Fig. 6B). More importantly, the intratumoral injection of CCL18 into xenografts formed by MCF-7 cells co-implanted with NBFs dramatically reduced breast cancer cell apoptosis and sustained tumor growth in mice receiving chemotherapy (Fig. 6D, E and Supplementary Fig. 6C, D). However, CCL18 administration to xenografts formed by MCF-7 cells alone did not influence the effects of chemotherapy or tumor growth in vivo (Fig. 6D, E and Supplementary Fig. 6C, D). Collectively, these data suggested that CD10^+^GPR77^+^ fibroblasts induced by CCL18 generate a niche for CSC enrichment, leading to the chemoresistance of tumor cells in vivo.

To explore the possibility of CCL18 as a therapeutic target, we co-inoculated MCF-7 cells with or without NBFs and TAMs into the mammary fat pads of NOD/SCID mice and blocked CCL18 signaling by anti-CCL18 neutralizing antibody. When the tumors became palpable, the mice received chemotherapy. Determined by tumor volume (Fig. 6F and Supplementary Fig. 6E) and the tumor cell apoptosis (TUNEL^+^EPCAM^+^) (Fig. 6G, H), we found that co-injection of TAMs, NBFs and tumor cells MCF-7 dramatically enhanced the chemoresistance of MCF-7 cells, compared with the mice co-injected with MCF-7 cells and NBFs or MCF-7 cells and TAMs. Moreover, blocking CCL18 signaling significantly recovered the chemotherapeutic sensitivity of xenografts formed by co-injection of NBFs, TAMs and MCF-7 cells, but not the xenografts formed by co-injection of TAMs and MCF-7 cells (Fig. 6F–H and Supplementary Fig. 6E). Furthermore, immunostaining showed that CCL18^+^ TAMs were abundantly infiltrated in the mice co-injected with MCF-7 and TAMs, with or without NBFs, but only the mice injected with NBFs, MCF-7 cells and TAMs had an abundant enrichment of chemoresistance-inducing CD10^+^GPR77^+^ CAFs (Fig. 6G, H). In addition, the number of CD10^+^GPR77^+^ CAFs were significantly reduced in the mice injected with anti-CCL18 neutrilizing antibody. These data suggest that targeting CCL18 could inhibit the formation of CD10^+^GPR77^+^ CAFs, and recover the chemosensitivity, leading to effective tumor control.

## Discussion

CAFs are one of the most abundant stromal cells in the tumor microenvironment and have prominent roles in cancer progression, including remodeling the ECM for tumor invasion [34], providing a niche to support chemoresistance [3], secreting cytokines to promote angiogenesis [1] and inducing immunosuppression [8]. However, CAFs are not a homogenous population, and their considerable heterogeneity greatly hinders the development of targeted therapies [5, 6]. Emerging evidence suggests that different cellular origins and inducers of cell activation may be responsible for the heterogeneity of CAFs [33, 46]. In our study, we found that a chemoresistance-inducing CAF subpopulation, CD10^+^GPR77^+^ CAF, was derived from NBFs and activated by the TAM-secreting CCL18. TAMs, the most abundant inflammatory cells in the tumor microenvironment [47], are key orchestrators of tumor-associated inflammation, directly affecting neoplastic cell growth [48], neoangiogenesis [49, 50], and ECM remodeling [51]. In the studies by our group and others, a large number of TAMs were observed in the vicinity of areas with abundant CAFs, the largest component of the tumor stroma, suggesting close crosstalk between these two cell types. In hepatocellular carcinoma, p53-deficient hepatic stellate cells, which are similar to fibroblasts, were shown to mediate the M2 polarization of macrophages and were associated with immunosuppression in a mouse model [52]. In prostate cancer, CAFs can recruit and activate monocytes to generate M2 macrophages via CXCL12 and CXCL14 [53, 54]. Reciprocally, TAMs activate CAFs to promote tumor progression [55]. All these studies have revealed that the interaction between CAFs and TAMs may be largely responsible for the considerable heterogeneity and plasticity of the tumor microenvironment. However, our present study advanced the field by demonstrating that TAMs play a critical role in activating NBFs to become a specific subset of CAFs, thereby promoting malignancy. This finding highlights the significance of reciprocal interactions between different stromal cell types in tumor progression, beyond direct interactions between malignant and stromal cells. Moreover, to our knowledge, this is the first study to illustrate the mechanism that initiates the activation of a specific CAF subset.

NF-κB signaling is one of the most important inflammatory pathways in cancer development, not only in cancer cells but also in infiltrating stromal cells. During incipient neoplasia, dermal fibroblasts can be educated and transformed into CAFs by IL-1β-triggered NF-κB activation [23]. Consistently, our previous study showed that persistent NF-κB activation with sustained p65 nuclear retention in CD10^+^GPR77^+^ CAFs is essential for these cells to maintain their functional phenotype. We further revealed that the p65 acetylation responsible for sustaining NF-κB activation in CD10^+^GPR77^+^ CAFs stemmed from the self-production of C5a and was independent of IKK or IκB activity [3]. However, the mediator responsible for initiating NF-κB activation in fibroblasts is still unknown. Here, we demonstrated that CCL18 dramatically induced the phosphorylation of IKK and IκB in breast fibroblasts, leading to the subsequent abundant production of IL-6 and IL-8, as well as the overexpression of the functional surface markers CD10 and GPR77. When GPR77 was upregulated in fibroblasts, the complement signaling pathways kicked in to maintain NF-κB activation and the phenotype of CD10^+^GRP77^+^ CAFs. On the contrary, the TGF-β-induced myofibroblast transition of NBFs did not involve the generation of a chemoresistance-inducing phenotype. TGF-β signaling derived from cancer cells or stromal cells initiates the differentiation of fibroblasts into myofibroblasts by inducing SMAD2 activity and contributes to malignant progression, especially distant metastasis [31, 56]. Thus, our findings emphasize the mediator and corresponding signaling pathway responsible for initiating the activation of a specific functional CAF subset, revealing that different inflammatory factors in the tumor microenvironment may be responsible for the high plasticity and heterogeneity of CAFs [33, 46].

It has been well established that PITPNM3 is a functional receptor of CCL18 in breast tumor cells and T lymphocytes [29, 40]. PITPNM3 mediates the effects of CCL18 in two contexts. In cancer cells, CCL18–PITPNM3 enhances migration and EMT [29, 42, 57], but does not enhance the survival of breast tumor cells under chemotherapy. Although CCL18 induces the increased vimentin and decreased E-cadherin, as well as the upregulation of GM-CSF, IL-8, CCL2, and GRO [42], the upregulated levels of IL-8 in breast cancer cell might be not enough to induce the enrichment of cancer stem cell and mediate chemoresistance. By contrast, CCL18-PITPNM3 signaling in stromal cells might cause the explosive release of inflammatory cytokines, which will influence the characteristics of cancer cells. In fibroblasts, CCL18 activates NF-κB pathway and produced a tremendous amount of IL-6 and IL-8, which induce the stemness and chemoresistance of cancer cells. The differential expression of NF-κB target genes in different cell types might be contributed to the distinct transcriptional activities regulated by epigenetics, which needs further investigation. Furthermore, CCL18-PITPNM3 signaling also plays other roles in breast tumor progression, such as immunosuppression and angiogenesis. Tumor-infiltrating naive CD4^+^ T cells are recruited to breast tumors by CCL18 and converted to functional immunosuppressive Tregs [40]. In addition, CCL18 induces EndoMT, which activates ERK and Akt/GSK-3β/Snail signaling in endothelial cells, thereby contributing to proangiogenic effects [58]. Furthermore, CCL18 induces myofibroblast differentiation and promotes the proliferation and invasion of Phyllodes tumor cells [59]. In conclusion, CCL18 signaling from TAMs facilitates a protumorigenic microenvironment through PITPNM3. Upon the binding of CCL18 to PITPNM3, Pyk2 is phosphorylated and translocates from the cytoplasm to the plasma membrane to form a stable complex with PITPNM3, which subsequently activates Src kinase and triggers integrin alpha5/beta1 clustering [60] and NFκB activation [61]. PITPNM3 knockdown with CD4-aptamer-PITPNM3-siRNA chimeras in naive CD4^+^ T cells in a humanized mouse tumor model significantly attenuated primary tumor cell survival in situ and lung metastasis by promoting antitumor immunity [40]. Here, PITPNM3 knockdown in NBFs dramatically impeded the CCL18-induced activation of fibroblasts. Blocking CCL18 by administration of anti-CCL18 neutralizing antibody could inhibit the formation of CD10^+^GPR77^+^ CAFs in vivo, and recover the chemosensitivity, leading to effective tumor control. Our findings further expand our knowledge of extensive PITPNM3 expression and its functional role in the tumor microenvironment, suggesting that CCL18-PITPNM3 could be an attractive therapeutic target in the tumor microenvironment. Blocking CCL18–PITPNM3 signaling could not only impede tumor cell metastasis, reverse immunosuppression, suppress angiogenesis and inhibit tumor progression but also block the evolution of tumor-promoting CAFs in the early stage of tumor progression.

## Methods

### Patients and samples

Primary invasive breast carcinoma tissues were obtained from 259 patients at Sun Yat-Sen Memorial Hospital, Sun Yat-Sen University, between 2006 and 2019. All patients received neoadjuvant chemotherapy after a definitive diagnosis using puncture specimens, and the chemotherapy regimens were as follows: four cycles of AC (doxorubicin 60 mg/m^2^ plus cyclophosphamide 600 mg/m^2^) every 3 weeks and paclitaxel (80 mg/m^2^) weekly for 12 weeks or four cycles of TC (docetaxel 75 mg/m^2^ plus cyclophosphamide 600 mg/m^2^) every three weeks. RECIST (Response Evaluation Criteria in Solid Tumors) was used to evaluate therapeutic efficacy. Patients with a complete response (CR) and a partial response (PR) were classified as chemotherapy-sensitive, while those with stable disease (SD) and progressive disease (PD) were classified as chemotherapy-resistant. The scRNA-seq data from 50 breast cancer specimens and 13 normal breast samples were obtained from GEO: GSE161529 and the Pancancer TME Blueprint (https://lambrechtslab.sites.vib.be/en/pan-cancer-blueprint-tumour-microenvironment-0).

### Primary cell and tissue culture

Primary normal breast fibroblasts (NBFs) were isolated from reduction mammaplasty samples, and primary cancer-associated fibroblasts (CAFs), tumor-associated macrophages (TAMs) and T lymphocytes were isolated from treatment-naive breast carcinoma samples obtained during breast cancer resection. Briefly, tissues were cut into fragments of approximately 1 mm^3^ and then digested by enzymatic hydrolysis (DMEM supplemented with 10% FBS, 1.5 mg/mL collagenase type I, 1.5 mg/mL collagenase type III and 1.5 mg/mL hyaluronidase) at 37 °C with gentle agitation for the indicated time (2.5 h for fibroblasts and 1 h for TAMs). The dissociated tissues were resuspended and filtered through a 70-μm cell strainer to obtain a cell suspension, which was centrifuged at 250 x *g* for 5 min to acquire the stromal fraction. Fibroblasts were further purified using anti-fibroblast microbeads (Miltenyi Biotec, Cat.No.130-050-601). To isolate TAMs and T lymphocytes, the primary cell suspension was centrifuged at 400 × *g* for 5 min, and then, CD14-positive cells or T cells were further purified by using CD14 microbeads (Miltenyi Biotec, Cat.No.130-050-201) or a Pan T Cell Isolation Kit (Miltenyi Biotec, Cat.No.130-096-535). After verification [3, 40, 42], the isolated primary fibroblasts and TAMs were cultured in DMEM supplemented with 10% FBS, and T lymphocytes were cultured in RPMI-1640 supplemented with 10% FBS. Primary fibroblasts at passages 1–5 were used in our experiments. In some experiments, fibroblasts were cultured with rCCL18 (20 ng/mL, # 300-34, PeproTech), rTGF-β (10 ng/mL, # 100-21, PeproTech) or the indicated conditioned medium (CM) for 7 days. The concentration of CCL18 used to treat cells was set based on the concentration of CCL18 that secreted by TAMs detected by us or others [29, 42, 62]. For gene silencing, fibroblasts were transduced with LV3 lentivirus carrying target gene-specific shRNA constructs (Supplementary Table 1). Lentivirus was provided by Gene Pharma Inc. (Shanghai, China). To inhibit specific signaling pathways, fibroblasts were pretreated with vehicle (DMSO), JSH-23 (6 mM, Selleck, #S7351) or Bay11-7082 (2 mM, Selleck, #S2913). TAMs were pretreated with IgG, TGF-β neutralizing antibody (10 µg/mL, R&D, #MAB1835) or CCL18 neutralizing antibody (10 µg/mL, R&D, #AF394). Tumor CM was supplemented with CCL18 neutralizing antibody, IL6 neutralizing antibody (10 µg/mL, BD, #554543) or IL8 neutralizing antibody (10 µg/mL, BD, #554726) prior to the experiments.

To harvest tumor CM, treatment-naive breast carcinoma samples were cut into fragments of approximately 1 mm^3^ and then placed on top of sponges in a 12-well cell culture dish. Then, all the tissues were cultured with explant medium (DMEM supplemented with 10% FBS, 10 µg/mL insulin and 5 µg/mL hydrocortisone) at 37 °C and 5% CO_2_ for 36 h.

All samples were collected from patients who provided written informed consent, and the related protocols were reviewed and approved by the Ethics Committee of Sun Yat-Sen Memorial Hospital.

### Western blot

Cells were lysed in RIPA buffer (Millipore) supplemented with protease and phosphatase inhibitors (Thermo Fisher Scientific, #78444), and the proteins were collected by centrifugation at 4 °C. A Pierce BCA Protein Assay kit (Cat# 23225, Thermo Fisher Scientific) was used for protein quantification. Equivalent amounts of protein from each sample were resolved by 10% SDS–PAGE and then transferred to PVDF membranes, which were then probed with primary antibodies against α-SMA (1:1000, R&D, #MAB1420), FAP (1:500, R&D, #AF3715), CD10(1:1000, Abcam, #ab255609), GPR77((1:1000, Abcam, #ab96808), caspase-3 (1:1000, CST, #9662), cleaved caspase-3 (1:1000, CST, #9664), PARP (1:1000, CST, #9532), cleaved PARP (1:1000, CST, #5625), phospho-IKKα/β (1:500, CST, #2697), IKKα (1:1000, CST, #2682), IKKβ (1:1000, CST, #2678), SMAD2/3 (1:1000, CST, #8685), phospho-SMAD2 (1:1000, CST, #18338), phospho-SMAD3 (1:1000, CST, #9520), PITPNM3 (1:1000, Novus Biologicals, #NBP1-31070), and GAPDH (1:1000, Proteintech, #HRP-60004), followed by incubation with an HRP-linked secondary antibody (CST). The antigen–antibody reactions were visualized by chemiluminescence-based immunodetection (ECL, Thermo).

### Flow cytometry

To detect cell surface markers, cells suspended in PBS containing 1% FBS and 2 mM EDTA were treated with FcR blocking reagent (Miltenyi Biotec, #130-059-901) and then incubated with specific fluorescence-linked antibodies against CD10 (BioLegend, #312204), GPR77 (BioLegend, #342406), CD44 (BD Biosciences, #555478), CD24 (BD Biosciences, #55542), CCR6 (BioLegend, #353409) and CCR8 (BioLegend, #360603) for 30 min at 4 °C. To evaluate cell surface PITPNM3 expression, cells were incubated at 4 °C with anti-PITPNM3 primary antibody (Novus Biologicals, #NBP1-31070) for 60 min and then incubated with a fluorescein-conjugated secondary anti-rabbit IgG antibody (Jackson ImmunoResearch, Bar Harbor, ME) at 4 °C for 45 min. In addition, to detect ALDH1 activity, an ALDEFLUOR kit was used according to the manufacturer’s instructions (Stem Cell Technologies, #01700). To detect apoptosis, tumor cells with indicated treatment were dissociated using 0.25% trypsin-EDTA and then harvested by centrifugation. Cell apoptosis was assessed by using the Annexin V Apoptosis Detection Kit (eBioscience, #88-8005-74). Briefly, cells were incubated with 5 µL FITC-conjugated Annexin V antibody in 100 μL binding buffer at room temperature for 15 min. Then, the cells were rinsed and resuspended in 200 μL buffer supplemented with 5 μL propidium iodide. Specimens were subsequently analyzed on a BD Accuri C6 or Thermo Attune NxT flow cytometer.

### Immunofluorescence

For immunostaining of paraffin sections, the sections were deparaffinized, and antigen retrieval was then performed in 0.01 M citrate buffer (pH 6.0). For immunostaining of cultured cells, the cells were fixed with paraformaldehyde and then permeabilized with 0.1% Triton X-100 on ice for 15 min. Nonspecific antigen epitope binding was blocked by incubation with phosphate buffer containing 5% BSA for 1 h. Next, sections or cells were probed overnight at 4 °C with specific primary antibodies, including goat anti-human α-SMA (Abcam, # ab21027, 1:100), rabbit anti-human α-SMA (Abcam, # ab124964, 1:100), mouse anti-human ALDH1 (R&D, # AF5869, 1:100), rabbit anti-human CD10 (Abcam, # ab73409, 1:30), mouse anti-human GPR77 (BioLegend, #342402, 1:30), sheep anti-human FAP (R&D, # AF3715, 1:100), rabbit anti-human CD68 (Abcam, # ab213363, 1:100), goat anti-human CCL18 (R&D, # AF394, 1:100), rabbit anti-human p65 (CST, # 8242, 1:50), rabbit anti-human ac-p65 (Abcam, # ab19870, 1:50), goat anti-human IL6 (R&D, # AF-206, 10 µg/mL) and mouse anti-human IL8 (R&D, # MAB208, 20 µg/mL). Then, antigen–antibody binding was visualized by using Alexa Fluor-conjugated secondary antibodies (Invitrogen) according to the manufacturer’s instructions. DAPI (# D3571, Thermo Fisher Scientific) was used for nuclear counterstaining, and laser scanning confocal microscopy (LSM780, Zeiss) was used for imaging. Staining cells quantification was counted in at least five fields per section and the mean of counts was used for statistical analysis.

### Cytokine antibody arrays

The cytokine profile of tissue culture medium was determined by using a Human Cytokine Antibody Array V kit (RayBiotech). Briefly, membrane arrays were incubated with 1 mL tissue culture medium overnight at 4 °C, and the next day, biotin-linked antibodies were used to create an antibody–antigen–antibody sandwich. Next, an HRP-conjugated secondary antibody was used to amplify the signal, which was detected by incubation with a chemiluminescent substrate and X-ray exposure. Quantitative analysis was conducted with Array Vision Evaluation 8.0 (GE Healthcare Life Science).

### ELISA

Fresh breast cancer puncture specimens or cells exposed to the indicated treatment were cultured in DMEM supplemented with 10% FBS for 24 h, and the supernatant was collected by centrifugation for subsequent ELISA analysis. IL-6 (eBioscience Cat# 88-7066-86), IL-8 (eBioscience Cat# 88-8086-86) and CCL18 (R&D Systems Cat #DCL180B) ELISA kits were used according to the manufacturers’ instructions.

### Immunohistochemistry

Paraffin sections were deparaffinized, and then, antigen retrieval was performed in 0.01 M citrate buffer (pH 6.0). The sections were then incubated overnight at 4 °C with a primary antibody against CCL18 (1:100, R&D, #AF394), and the signal was amplified with an HRP-conjugated secondary antibody and visualized using DAB (Dako). Upright metallurgical microscope (BX53, Olympus) was used for imaging. Staining cells quantification was counted in at least five fields per section and the mean of counts was used for statistical analysis and the calculation and the statistical analysis was performed by two independent researchers.

### scRNA-seq transcriptome data processing

Multiple breast samples from GEO series GSE161529 and the Pancancer TME Blueprint (https://lambrechtslab.sites.vib.be/en/pan-cancer-blueprint-tumour-microenvironment-0) were combined using the Seurat analysis package. Conservative quality control cutoffs were set according to the number of genes/cell (>500) and the percentage of mitochondrial unique molecular identifier (UMI) counts (<20%). Unless otherwise stated, the cluster resolution was set to 0.5 for t-distributed stochastic neighbor embedding (t-SNE). EPCAM-negative nonepithelial cells were subjected to further regrouping, and cell clusters in the microenvironment were annotated based on canonical cell type markers for fibroblasts (PDGFRA, PDGFRB, ACTA2, PDPN and FAP), macrophages (CD14 and CD68), endothelial cells (VWF), T cells (CD3D, CD8A and CD4), B cells (CD79A) and pericytes (NG2). The positive threshold was set as log expression level >0.5 for subsequent analysis.

### Collagen gel contraction assay

The collagen gel contraction assay was a widely-used and classical functional assay used to examine the activation status of fibroblasts [35]. To evaluate NBF function, NBFs were treated with indicated cytokine or cocultured with primary TAMs in an indirect transwell coculture system. Then, NBFs were harvested by trypsin digestion and a mixture of 1.0 × 10^4^ fibroblasts and 200 µl collagen mix was plated in 24-well culture dishes. The gel was allowed to polymerize for 30 min at 37 °C, after which 500 μL medium was added to the wells, and a sterile pipette tip was used to detach the gels from the well walls. The wells were imaged after 24 h to measure the gel dimensions compared to the well diameter using ImageJ software.

### RT–qPCR

Total RNA was extracted from cultured cells by using TRIzol Reagent (Invitrogen). Then, quantitative reverse transcription PCR (qRT–PCR) was performed using a SYBR Premix Ex Taq kit (TaKaRa, Japan) according to the manufacturer’s instructions, and data were collected and analyzed with a LightCycler 480 instrument (Roche). The primer sequences are listed in Supplementary Table 2.

### Co-culture experiments

MCF-7, BT474, BT549 and SKBR3 breast cancer cells were obtained from American Type Culture Collection (ATCC) and tested regularly for Mycoplasma infection. A six-well Transwell apparatus with a 0.4 µm pore size (Corning Incorporated) was used for the coculture experiments. A total of 5 × 10^4^ cancer cells were seeded in each upper chamber, and 5 × 10^4^ pretreated fibroblasts were seeded in each lower chamber. The cocultured cells were passaged when they reached 90% confluence.

### MTT assay

A 3-(4,5-dimethylthiazol-2-yl)22,5-diphenyltetrazolium bromide (MTT, Sigma) assay was used to determine cell viability. Briefly, 1 × 10^3^ tumor cells/well were plated in 96-well plates and then treated with the indicated chemotherapy agent for the indicated time. Then, MTT solution was added, and the plates were incubated at 37 °C for 4 h. Thereafter, the media were removed, and the formazan crystals were dissolved in DMSO (150 mL/well). Then, the absorbance was measured at 540 nm using an Infinite F500 (Tecan).

### Sphere formation assay

Tumor cells (1 × 10^3^ cells/mL) were cultured in DMEM-F12 (GIBCO) supplemented with 20 ng/mL EGF, B27 (1:50, Invitrogen), 4 mg/mL insulin, and 0.4% BSA in 6-well ultralow adhesion plates for two weeks. Then, cell spheres with a diameter >75 mm were counted.

### Fluorescence-activated cell sorting

Using a BD Influx flow cytometer, different subsets of CAFs (CD10^+^GPR77^+^ and CD10^+^GPR77^+^-depleted) were selected by fluorescence-activated cell sorting (FACS). Before cell sorting, primary CAFs were resuspended in PBS containing 1% FBS and incubated with antibodies against CD10 and GPR77 for 30 min at 4 °C. The purity of the sorted populations was verified by flow cytometry.

### Luciferase reporter assay

pNF-κB-Luc, pRL-TK, and pTAL-Luc vectors (Promega Madison, WI) were transfected into cells using Lipofectamine 3000 (Invitrogen, Carlsbad, CA). The luciferase activity of transfected cells treated as indicated was determined with the Dual-Luciferase Reporter Assay System (Promega). Firefly luciferase activity was normalized to Renilla luciferase activity for each sample.

### TUNEL assay

Paraffin sections were deparaffinized, and then, cell death was detected by using the In Situ Cell Death Detection Kit POD (Cat# 11684817910, Roche) according to the manufacturer’s instructions.

### Coinjection animal experiments

Sample size and number of animals were chosen based on our prior experience [3] to achieve statistical significance. No mice were excluded from any analyses and no blind analysis was performed. All procedures were approved by the Institutional Review Boards and Animal Care and Use Committees of Sun Yat-Sen University.

### Tumorigenesis

Female NOD/SCID mice were randomly selected to each group (12 mice per group). Model A: serial concentrations of MCF-7 cells alone or mixed with primary NBFs were coinjected into the mammary fat pads of 6-weeks-old NOD/SCID mice at a ratio of 1:3 as previously described [1]. After cell injection, the mice received biweekly intratumoral injections of PBS or rCCL18 (0.1 mg/kg, Cat# 300-34, PeproTech). Tumor formation was assessed weekly for up to 3 months.

### Chemoresistance

Female NOD/SCID mice were randomly selected to each group (8 mice per group). The MCF-7 xenograft mouse model was generated as described above (model A) or generated by co-inoculating MCF-7 cells with or without NBFs and TAMs into the mammary fat pads of NOD/SCID mice (model B). In model B, MCF-7 cells were treated with 10 ng/ml TNF-α for 2 weeks as described in our previous study [42] and PBMC were cultured with the CM of treated MCF-7 to induce TAMs before co-inoculating. After cell injection, the mice in model A received PBS or rCCL18 as described above and the mice in model B received IgG or anti-CCL18 neutralizing antibody (2 mg/kg, R&D, #AF394) weekly by intratumoral injecting. When the tumors reached 3 mm in diameter, 10 mg/kg docetaxel was administered by intraperitoneal injection once per week for 6 weeks.

The xenografts were harvested, fixed in 10% formaldehyde solution, and embedded in paraffin for further analysis. Tumor size was assessed weekly by calipers, and tumor volume was determined according to the standard formula: Volume (mm^3^) = (length × height^2^)/2.

### Statistical analysis

The detailed statistical analysis results are indicated in the figure legends or methods. Unless otherwise described in the figure legends or methods, the statistical analyses were performed using GraphPad Prism 8.0. Pearson correlation was used to assess the association between the infiltration of CCL18^+^ TAMs and CD10^+^GPR77^+^ CAFs. The number of CCL18^+^ TAMs and CD10^+^GPR77^+^ CAFs in 259 cases coincided with normal distribution. All in vitro experiments were repeated at least three independent times, and the specific number of experiments is indicated in the figure legends. The two-tailed Student’s *t* test was used to identify significant differences between two groups, and the two-tailed one-way ANOVA with Dunnett’s test was used to determine significant differences in experiments with more than two groups. Estimating of variation within each group was performed before comparation. The variance similar between groups was statistically compared. All bar graphs show means and error bars (indicating the standard error of the mean (SEM) or the standard deviation (SD)), as mentioned in each figure legend. *P* < 0.05 was considered to indicate statistical significance. No blind analysis was performed in this study.

## Supplementary information

Supplementary Figures and Tables

email responses from co-authors

## Data Availability

This study constituted a reanalysis of scRNA-seq transcriptome data. The datasets used in this study are available at the following sites: GEO series GSE161529 (https://www.ncbi.nlm.nih.gov/geo/) and the Pancancer TME Blueprint (https://lambrechtslab.sites.vib.be/en/pan-cancer-blueprint-tumour-microenvironment-0).
