# Supplementary material for: CCL18 signaling from tumor-associated macrophages activates fibroblasts to adopt a chemoresistance-inducing phenotype
Source: Oncogene. 2022 Nov 22;42(3):224–37. doi: 10.1038/s41388-022-02540-2 (PMC9836934; doi:10.1038/s41388-022-02540-2)
Supplement: Supplementary file 1 — Supplementary Figures and Tables [file 41388_2022_2540_MOESM1_ESM.pdf]

CCL18 signaling from tumor-associated macrophages activates fibroblasts to adopt a chemoresistance-inducing phenotype

Wenfeng Zeng[1,2,3,#], Lixiong Xiong[4,#], Wei Wu[1,2,3,#], Shunrong Li[1,2,3], Jiang Liu[1,2,3], Linbing Yang[1,2,3], Liyan Lao[1,2,3], Penghan Huang[1,2,3], Mengmeng Zhang[5], Huiping Chen[1,2,3], Nanyan Miao[1,2,3], Zhirong Lin[1,2,3], Zifei Liu[1,2,3], Xinyu Yang[1,2,3], Jiayi Wang[1,2,3], Pei Wang[1,2,3], Erwei Song[1,2,3], Yandan Yao[1,2,3*], Yan Nie[1,2,3*], Jianing Chen[1,2,3*], Di Huang[1,2,3*]

Supplementary Figures

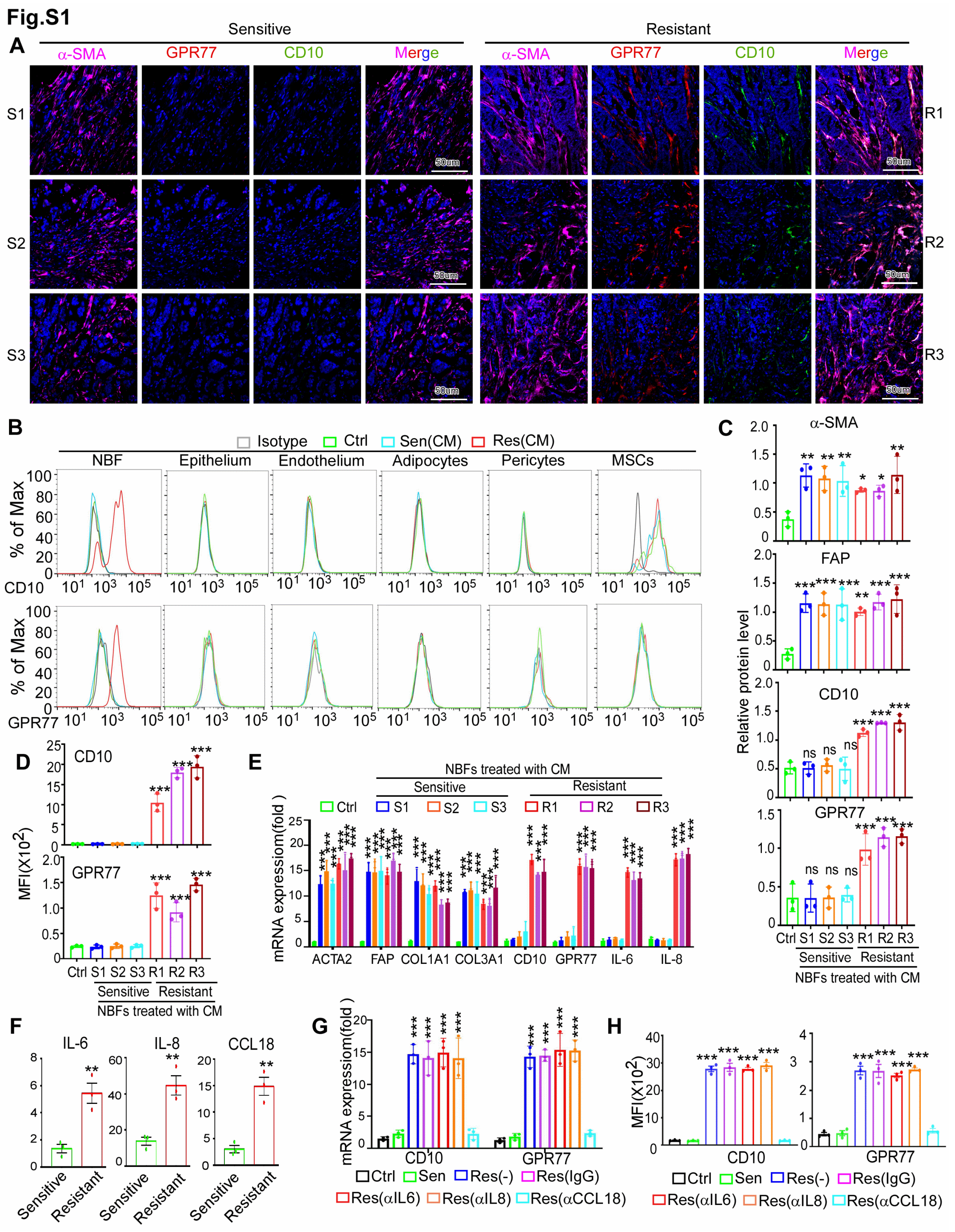

Figure S1. Breast cancers with different chemotherapeutic responses exhibit conspicuously distinct cytokine profiles in their tumor microenvironment, related to Figure 1

A, Representative images of immunofluorescent staining of α-SMA, CD10 and GPR77 in the pre-treatment breast cancer biopsies of chemosensitive (n=3) and chemoresistant (n=3) patients. Scale bars, 50 μm. **B,** Flow cytometric analysis for CD10 and GPR77 in primary normal NBFs, MSCs, pericytes, adipocytes, epithelial cells and endothelial cells treated with chemoresistant or chemosensitive tumor CM. **C,** Quantification of Fig.1B: relative α-SMA, FAP, CD10 and GPR77 protein levels in NBFs treated with chemoresistant (n=3) or chemosensitive (n=3) tumor CM was quantified using Image J. Protein levels were normalized to GAPDH. **D**, Quantification of Fig.1C: mean fluorescence intensity (MFI) of CD10 and GPR77 in NBFs treated with chemoresistant (n=3) or chemosensitive (n=3) tumor CM was quantified using Flowjo. **E**, QRT–PCR for ACTA2, FAP, COL1A1, COL3A1, CD10, GPR77, IL-6 and IL-8 in NBFs treated with chemoresistant (n=3) or chemosensitive (n=3) tumor CM. **F**, ELISA for IL-6, IL-8 and CCL18 levels in the CM of chemoresistant (n=3) and chemosensitive tumors (n=3). **G-H**, QRT–PCR(**G**) and mean fluorescence intensity (**H**) for CD10 and GPR77 in NBFs treated with chemosensitive tomor CM or chemoresistant tumor CM added without or with neutralizing antibodies against IL6, IL8 or CCL18.

Data expressed as mean ± SEM, *P<0.05, **P < 0.01 compared with chemosensitive tumor CM by two-tailed Student's t test **(F)**. Data expressed as mean ± SEM, *P< 0.05, **P < 0.01, ***P < 0.001 compared with untreated NBFs (Ctrl) by two-tailed one-way ANOVA with Dunnett's multiple comparison test **(C-E**, **G-H)**.

Fig.S2

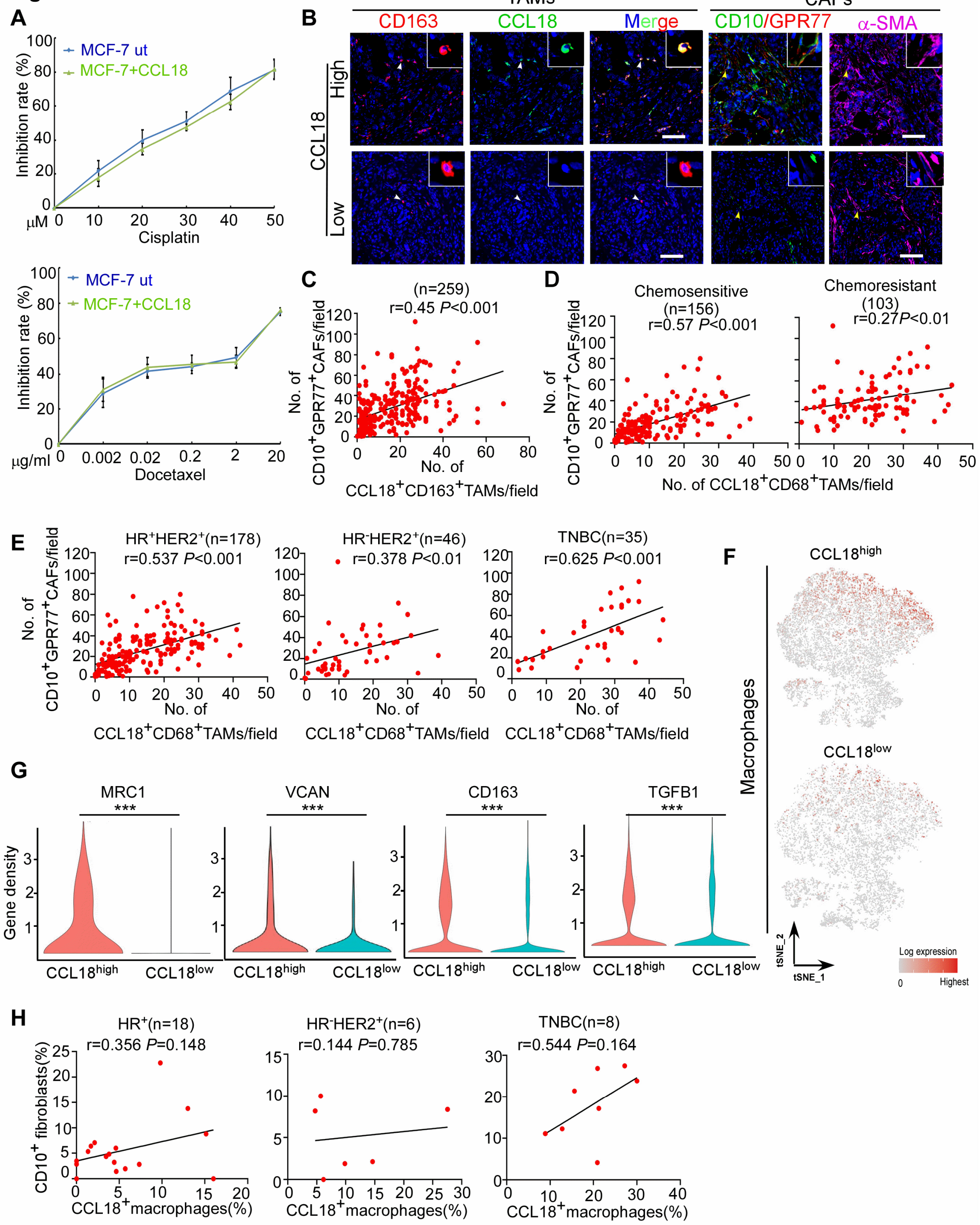

Figure S2. The intratumoral accumulation of CCL18⁺ tumor-associated macrophages is associated with the abundance of CD10⁺GPR77⁺ CAFs and chemoresistance, related to Figure 2

A, The growth inhibition rate of cisplatin (up) or docetaxel (down) on MCF-7 cells treated without or with CCL18 (n=3). Data expressed as mean ± SEM, P > 0.1 by two-

tailed Student's t test. **B**, Representative images of immunofluorescent staining of CD163, CCL18, CD10, GPR77 and α-SMA in serial sections of breast cancer samples with high or low CCL18 expression (n=259). Scale bars, 50 μm. The arrowheads denote the area of higher-magnification images shown in the top-right corner. **C**, The correlation between the abundance of $CD10^+GPR77^+$ CAFs and $CD163^+CCL18^+$ TAMs in breast cancer samples. The Pearson's correlation coefficient *r* value and *P* values were determined by two-tailed Pearson correlation coefficient test (n=259). **D-E**, Stratification analysis of the correlation between the abundance of $CD10^+GPR77^+$ CAFs and $CD68^+CCL18^+$ TAMs in breast tumors with different chemotherapeutic responses (**D**) and molecular subtypes(**E**). HR, hormone receptor. HER2, human epidermal growth factor receptor-2. TNBC, triple negative breast cancer. The Pearson's correlation coefficient r value and *P* values were determined by two-tailed Pearson correlation coefficient test. **F**, t-SNE layout of CCL18 expression in divided $CCL18^{high}$ and $CCL18^{low}$ patients from pancancer TME blueprint and GEO: GSE161529. **G**, Violin plots indicating the expression of MRC1, VCAN, CD163 and TGFB1 in macrophages of $CCL18^{high}$ or $CCL18^{low}$ patients. ***$P < 0.001$ compared with $CCL18^{low}$ patients by Student's t test. **H**, The correlation between the abundance of $CD10^+$fibroblasts and the infiltration of $CCL18^+$ macrophages in breast cancer with different molecular subtypes based on scRNA-seq transcriptomes from GEO: GSE161529. The Pearson's correlation coefficient r value and *P* values were determined by two-tailed Pearson correlation coefficient test.

Fig.S3

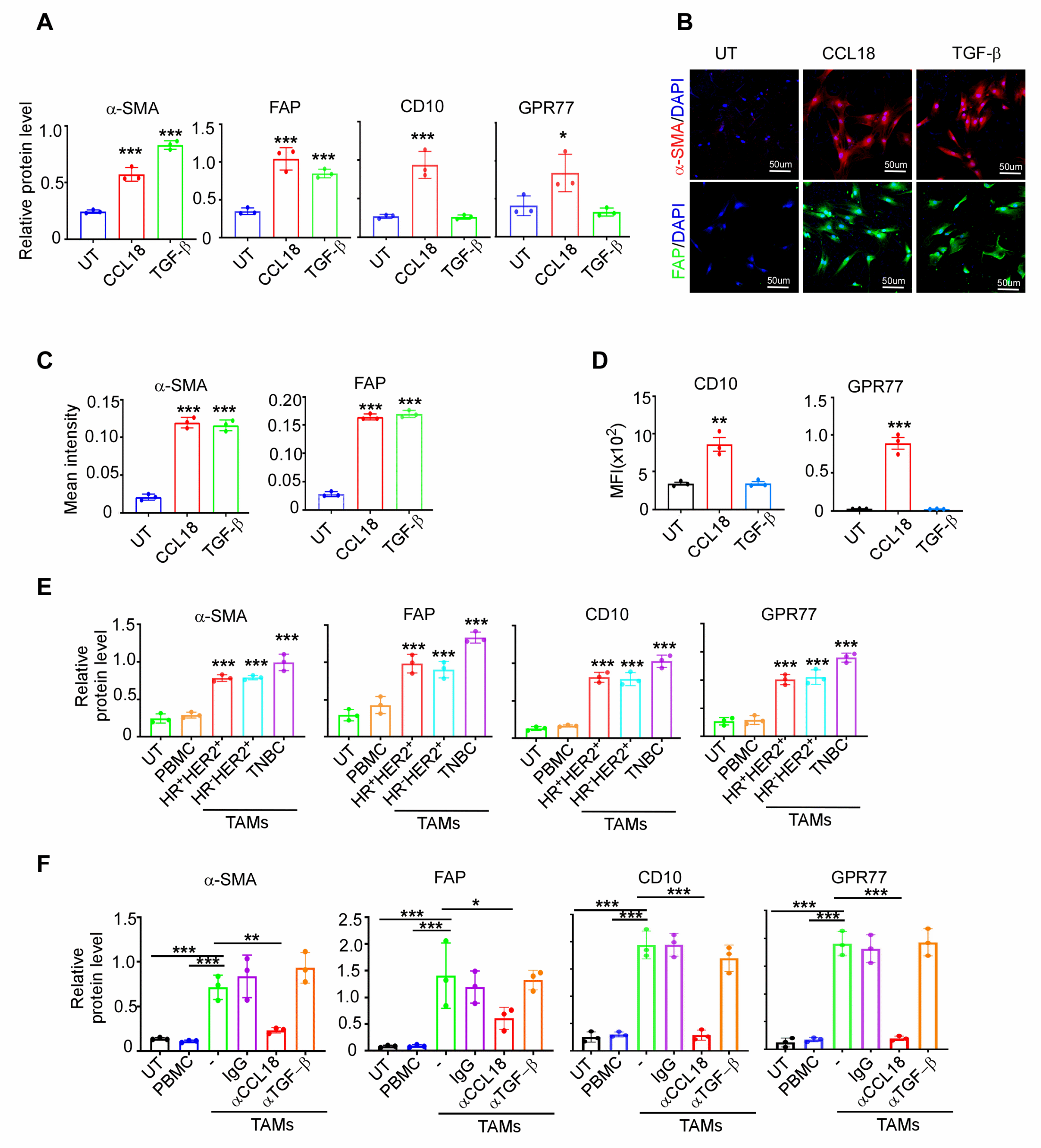

Figure S3. CCL18 produced by TAMs mediates the chemoresistance-inducing phenotype polarization in NBFs, related to Figure 3

A, Quantification of Fig.3C: relative α-SMA, FAP, CD10 and GPR77 protein levels in NBFs with indicated treatment were quantified using ImageJ (n=3). Protein levels were normalized to GAPDH. **B-C**, Representative immunofluorescent images(**B**) and mean fluorescent intensity(**C**) for α-SMA and FAP in NBFs with indicated treatment (n=3). Scale bars, 50 μm. **D**, Quantification of Fig.3D: mean fluorescence intensity for CD10

and GPR77 in NBFs with indicated treatment. **E**, Quantification of Fig.3E: relative α-SMA, FAP, CD10 and GPR77 protein levels in NBFs cultured alone (UT) or co-cultured with PBMC or TAMs isolated from different subtypes of breast cancer (n=3). **F**, Quantification of Fig.3F: relative α-SMA, FAP, CD10 and GPR77 protein levels in NBFs cultured alone (UT) or co-cultured with PBMC or TAMs pretreated with control IgG or neutralizing antibodies against CCL18 or TGF-β (n=3).

Data expressed as mean ± SEM, *P< 0.05, **P< 0.01, ***P < 0.001 compared with untreated NBFs **(A**, **C-E)** or NBFs co-cultured with untreated TAMs **(F)** by two-tailed one-way ANOVA with Dunnett's multiple comparison test.

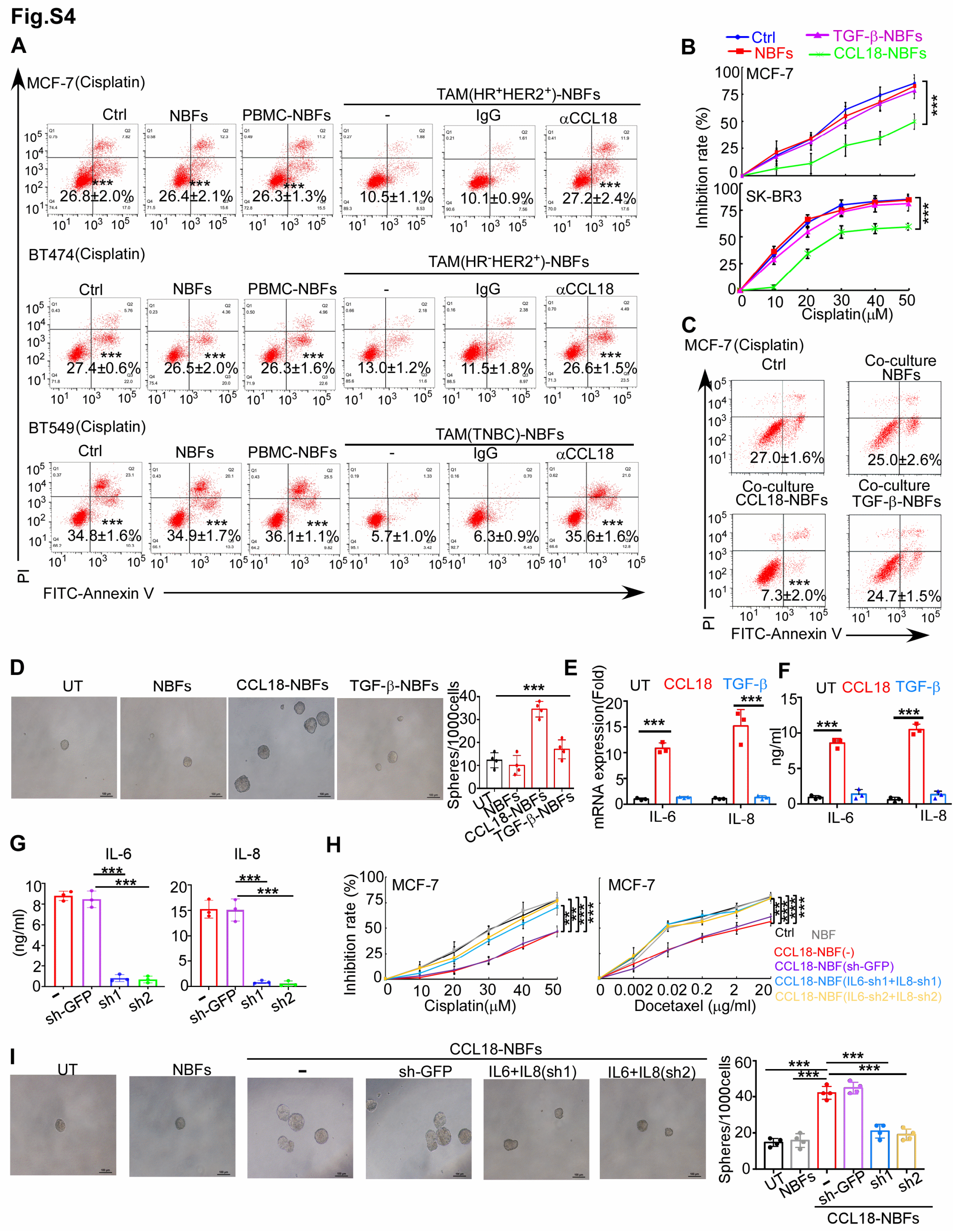

Figure S4. NBFs activated by CCL18-producing TAMs mediate chemoresistance and CSC enrichment by secreting IL-6 and IL-8, related to Figure 4

A, The proportion of apoptotic breast cancer cells treated with cisplatin, cultured alone (Ctrl) or co-cultured with the fibroblasts with indicated treatment. The proportion of Annexin V^{+}/PI^{-} (early apoptosis) and Annexin V^{+} /PI^{+} (late apoptosis) cells was shown

(n=3). **B**, The growth inhibition rate of cisplatin on MCF-7 (up) and SK-BR3 (down) cells cultured alone (Ctrl) or co-cultured with untreated or CCL18/TGF-β treated NBFs (n=3). **C**, The proportion of apoptotic MCF-7 cells, cultured alone (Ctrl) or co-cultured with indicated fibroblasts, challenged by cisplatin (n=3). **D**, Representative images (left) and quantification(right) of sphere formation in MCF-7 cultured alone or co-cultured with indicated NBFs (n=4). Scale bars, 100 μm. **E-F,** QRT–PCR(**E**) and ELISA(**F**) for IL-6 and IL-8 in NBFs with indicated treatment (n=3). **G**, ELISA for IL-6 and IL-8 in MCF-7 cultured alone or co-cultured with untreated or CCL18-treated NBFs transduced with shRNA against GFP, IL-6 and IL-8 (n=3). **H**, The growth inhibition rate of cisplatin (left) and docetaxel (right) on MCF-7 cultured alone or co-cultured with untreated or CCL18-treated NBFs transduced with shRNA against GFP, IL-6 and IL-8 (n=3). **I**, The sphere formation of MCF-7 cells cultured alone or co-cultured with NBFs treated as in **H** (n=3).

Data expressed as mean ± SEM, ***$P < 0.001$ compared with NBFs co-cultured with untreated TAMs(**A**), untreated NBFs **(B-F)** or CCL18-treated NBFs without transducing **(G-I)** by two-tailed one-way ANOVA with Dunnett's multiple comparison test.

Fig.S5

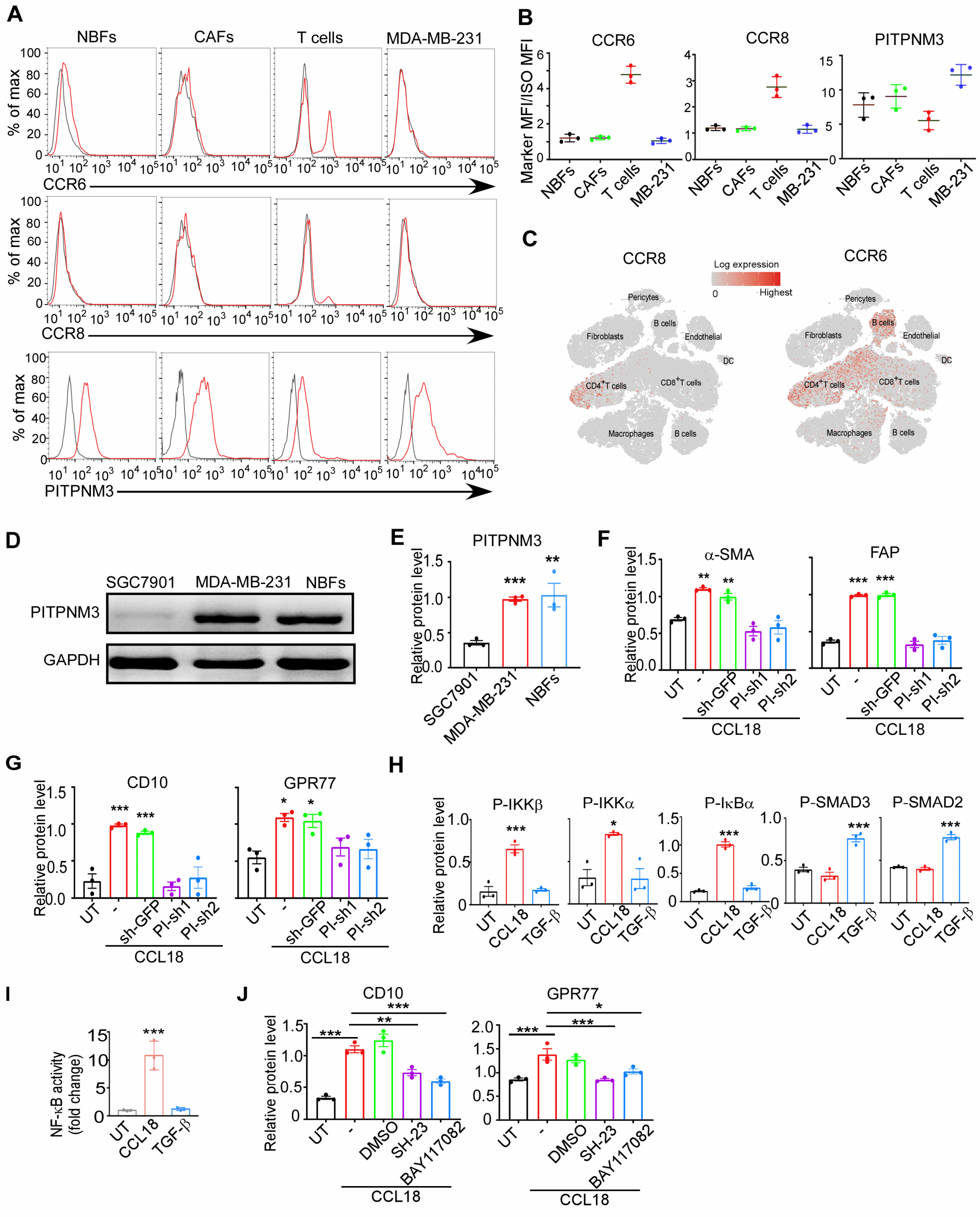

Figure S5. PITPNM3 mediates CCL18-induced fibroblast activation via NF-κB signaling, related to Figure 5

A-B, Representative flow cytometric analysis(**A**) and quantification of relative MFI (**B**) for CCR6, CCR8 and PITPNM3 in NBFs, CAFs, T cells and MDA-MB-231breast cancer cells(n=3). Grey line, isotype. Red line, CCR6/CCR8/PITPNM3. Relative MFI

were normalized using isotype MFI as loading control. **C**, Expression plots of CCR6 and CCR8 in microenvironment populations derived from scRNA-seq transcriptomes data were exhibited by t-SNE layout. **D-E**, Representative western blotting (**D**) and quantification (**E**) for the expression of PITPNM3 in NBFs, SGC7901 or MDA-MB-231 (n=3). **F-G**, Quantification of Fig.5A: relative α-SMA(**F**), FAP(**F**), CD10(**G**) and GPR77(**G**) protein levels in untreated or CCL18-treated NBFs transduced with GFP shRNA or PITPNM3 shRNAs (n=3). **H**, Quantification of Fig.5C: relative levels of phosphorylated IKKβ, IKKα, IκBα, SMAD2 and SMAD3 in NBFs with indicated treatment was quantified using ImageJ (n=3). Protein levels were normalized to GAPDH. **I**, Luciferase reporter assays for NF-κB activity in NBFs with indicated treatment (n=3). **J**, Quantification of Fig.5G: relative CD10 and GPR77 protein levels in untreated (UT) or CCL18-activated NBFs treated with DMSO, JSH-23 or BAY117082 (n=3).

Data expressed as Mean ± SEM, *P< 0.05, **P< 0.01, ***P < 0.001 compared with SGC7901(**E**), untreated NBFs**(F-I)** or untreated CCL18-activated NBFs **(J)** by two-tailed one-way ANOVA with Dunnett’s multiple comparison test.

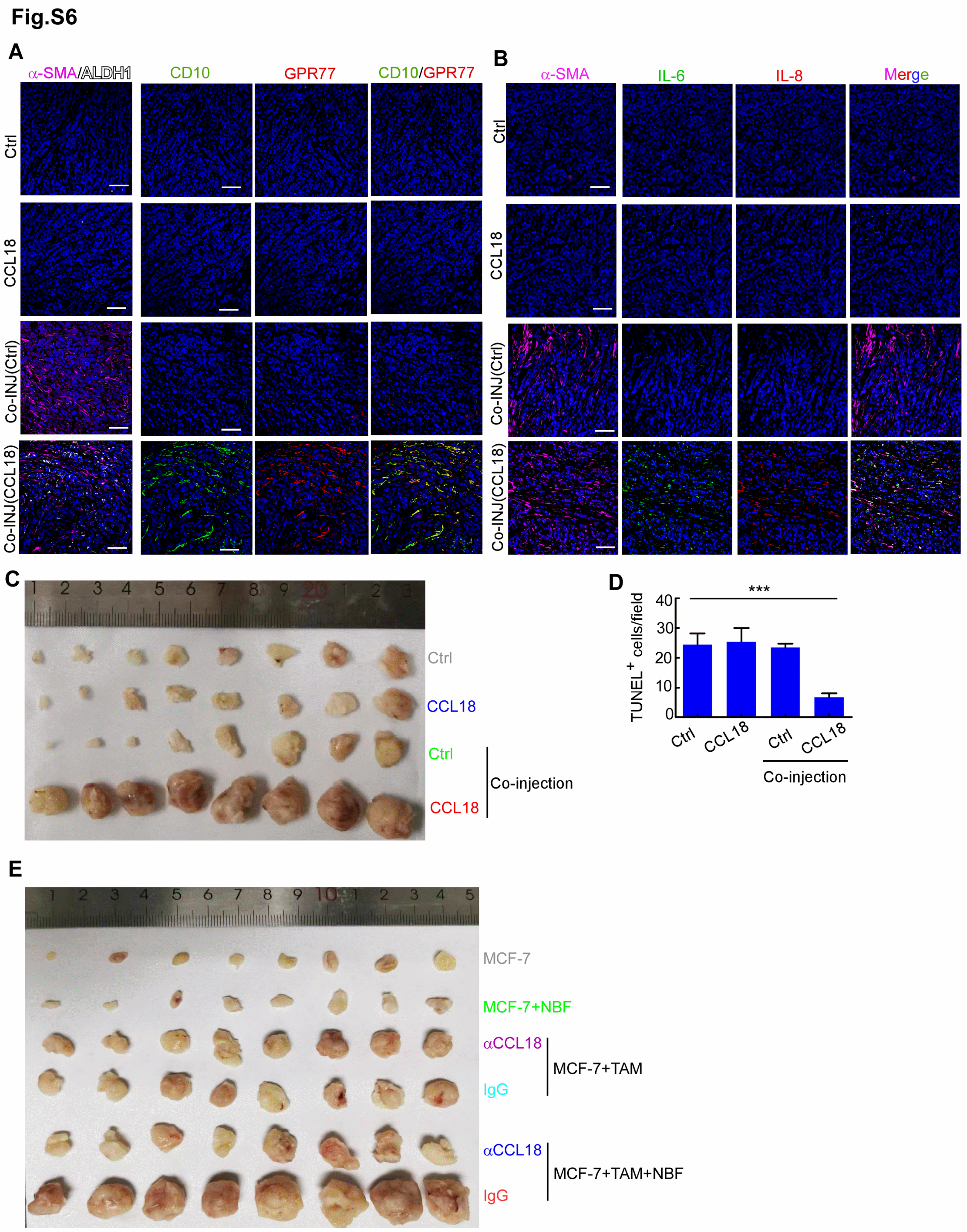

Figure S6. CCL18 promotes breast cancer tumorigenesis and chemoresistance in vivo by activating NBFs, related to Figure 6

A-B, Representative immunofluorescent images for (**A**) α-SMA, ALDH1, CD10 and GPR77, (**B**) α-SMA, IL6 and IL8 in the harvested xenografts related to Fig.6A-C. Scale bars, 50 μm. **C,** Picture of the harvested xenografts related to Fig.6D (n=8 per group).

D, Quantification for $TUNEL^+$ cells in the xenografts related to Fig.6E (mean ± SEM, n=8 per group). ***P < 0.001 by two-tailed one-way ANOVA with Dunnett's multiple comparison test. **E,** Picture of the harvested xenografts related to Fig.6F (n=8 per group).

Supplementary Tables

Supplementary Table 1. Targeting sequences for shRNA

Name	shRNA1	shRNA2
PITPNM3	5'-GGGAGAAGUGGCUUCGUAATT-3′	5′-CGCGCAUGAUCCUGCGCAATT-3′
P65	5'-GAGUCAGAUCAGCUCCUAA-3'	5'-GCUAUAACUCGCCUAGUGA-3'
IL-6	5'-CTTCCAATCTGGATTCAAT-3'	5'-CCCAGGAGAAGATTCCAAA-3'
IL-8	5'-GAAGAGGGCTGAGAATTCA-3'	5'-GCCAGATGCAATACAAGAT-3'

Supplementary Table 2. Primer sequences for qPT-PCR

**Name**	**Forward**	**Reverse**
CD10	5’-TGGATCTTGTAAGCAGCCTCA-3’	5’-GCACAACGTCTCCAAGTTGC-3’
GPR77	5’-CTGCTGACCATGTATGCCAG-3’	5’-CGCTGAACCGTAGACCACC-3’
ACTA2	5’-AAAAGACAGCTACGTGGGTGA-3’	5’-GCCATGTTCTATCGGGTACTTC-3’
FAP	5’ATGAGCTTCCTCGTCCAATTCA3’	5’AGACCACCAGAGAGCATATTTTG3’
COL1A1	5’GAGGGCCAAGACGAAGACATC3’	5’CAGATCACGTCATCGCACAAC3’
COL3A1	5’GGAGCTGGCTACTTCTCGC3’	5’GGGAACATCCTCCTTCAACAG3’
IL-6	5’-AAGCCAGAGCTGTGCAGATGAGTA-3’	5’-TGTCCTGCAGCCACTGGTTC-3’
IL-8	5’-ACACTGCGCCAACACAGAAATTA-3’	5’-TTTGCTTGAAGTTTCACTGGCATC-3’
GAPDH	5’-GGAGCGAGATCCCTCCAAAAT-3’	5’-GGCTGTTGTCATACTTCTCATGG-3’
