# Supplementary material for: CCL18 signaling from tumor-associated macrophages activates fibroblasts to adopt a chemoresistance-inducing phenotype
Source: Oncogene. 2022 Nov 22;42(3):224–37. doi: 10.1038/s41388-022-02540-2 (PMC9836934; doi:10.1038/s41388-022-02540-2)
Supplement: Supplementary file 2 — email responses from co-authors [file 41388_2022_2540_MOESM2_ESM.pdf]

Wenfeng Zeng

Reply | Reply All | Forward | Move | Mark | More | Delete

回复:(无主题)

2022-11-02 11:52:05

From : "曾文锋" <zengwf6@mail2.sysu.edu.cn>

To : "黄迪" <huangd63@mail.sysu.edu.cn>

I agree

--------------原始邮件--------------
发件人："黄迪 "<huangd63@mail.sysu.edu.cn>;
发送时间：2022年11月2日(星期三) 中午11:51
收件人："曾文锋" <zengwf6@mail2.sysu.edu.cn>;
主题：

Dear Dr Zeng,

We are planning to submit our manuscript entitled "CCL18 signaling from tumor-associated macrophages activates fibroblasts to adopt a chemoresistance-inducing phenotype" back to *Oncogene*. And our final author list is as below:

Wenfeng Zeng, Lixiong Xiong, Wei Wu, Shunrong Li, Jiang Liu, Linbing Yang, Liyan Lao, Mengmeng Zhang, Penghan Huang, Huiping Chen, Nanyan Miao, Zifei Liu, Xinyu Yang, Zhirong Lin, Jiayi Wang, Pei Wang, Erwei Song, Yan-Dan Yao, Yan Nie, Jianing Chen and Di huang

Please reply to my email confirming that you agree to these changes of the author list. And please feel free to contact us with any questions.

Look forward to hearing from you soon.

Best wishes.

Di Huang

Di Huang, PhD
Breast Tumor Center
Sun Yat-Sen Memorial Hospital

Lixiong Xiong

Reply | Reply All | Forward | Move | Mark | More | Delete

Re:

2022-11-02 15:13:04

From : "熊立雄" <xionglx3@mail.sysu.edu.cn>

To : "黄迪" <huangd63@mail.sysu.edu.cn>

agree

-----原始邮件-----
发件人:"黄迪" <huangd63@mail.sysu.edu.cn>
发送时间:2022-11-02 15:11:38 (星期三)
收件人: xionglx3@mail.sysu.edu.cn
抄送:
主题:

Dear Mr Xiong,

We are planning to submit our manuscript entitled "CCL18 signaling from tumor-associated macrophages activates fibroblasts to adopt a chemoresistance-inducing phenotype" back to *Oncogene*. And our final author list is as below:

Wenfeng Zeng, Lixiong Xiong, Wei Wu, Shunrong Li, Jiang Liu, Linbing Yang, Liyan Lao, Mengmeng Zhang, Penghan Huang, Huiping Chen, Nanyan Miao, Zifei Liu, Xinyu Yang, Zhirong Lin, Jiayi Wang, Pei Wang, Erwei Song, Yan-Dan Yao, Yan Nie, Jianing Chen and Di huang

Please reply to my email confirming that you agree to these changes of the author list. And please feel free to contact us with any questions.

Look forward to hearing from you soon.

Best wishes.

Di Huang

Di Huang, PhD
Breast Tumor Center
Sun Yat-Sen Memorial Hospital

Wei Wu

Reply | Reply All | Forward | Move | Mark | More | Delete

Re:

2022-11-02 17:59:18

From : "吴畏" <wuwei7@mail.sysu.edu.cn>

To : "黄迪" <huangd63@mail.sysu.edu.cn>

agree

-----原始邮件-----
发件人:"黄迪" <huangd63@mail.sysu.edu.cn>
发送时间:2022-11-02 14:10:40 (星期三)
收件人: wuwei7@mail.sysu.edu.cn
抄送:
主题:

Dear professor Wu,

We are planning to submit our manuscript entitled "CCL18 signaling from tumor-associated macrophages activates fibroblasts to adopt a chemoresistance-inducing phenotype" back to *Oncogene*. And our final author list is as below:

Wenfeng Zeng, Lixiong Xiong, Wei Wu, Shunrong Li, Jiang Liu, Linbing Yang, Liyan Lao, Mengmeng Zhang, Penghan Huang, Huiping Chen, Nanyan Miao, Zifei Liu, Xinyu Yang, Zhirong Lin, Jiayi Wang, Pei Wang, Erwei Song, Yan-Dan Yao， Yan Nie， Jianing Chen and Di huang

Please reply to my email confirming that you agree to these changes of the author list. And please feel free to contact us with any questions.

Look forward to hearing from you soon.

Best wishes.

Di Huang

Di Huang, PhD
Breast Tumor Center
Sun Yat-Sen Memorial Hospital

Shunrong Li

Subject: Re:
Agree

Li

在 2022年11月2日，14:27，LSR SYSU <lishr@mail.sysu.edu.cn> 写道:

发件人: 黄迪 <huangd63@mail.sysu.edu.cn>
日期: 2022年11月2日 GMT+8 14:21:04
收件人: lishr@mail.sysu.edu.cn

Dear Dr Li,

We are planning to submit our manuscript entitled "CCL18 signaling from tumor-associated macrophages activates fibroblasts to adopt a chemoresistance-inducing phenotype" back to *Oncogene*. And our final author list is as below：

Wenfeng Zeng, Lixiong Xiong, Wei Wu, Shunrong Li, Jiang Liu, Linbing Yang, Liyan Lao, Mengmeng Zhang, Penghan Huang, Huiping Chen, Nanyan Miao, Zifei Liu, Xinyu Yang, Zhirong Lin, Jiayi Wang, Pei Wang, Erwei Song, Yan-Dan Yao， Yan Nie， Jianing Chen and Di huang

Please reply to my email confirming that you agree to these changes of the author list. And please feel free to contact us with any questions.

Look forward to hearing from you soon.

Best wishes.

Di Huang

Di Huang, PhD
Breast Tumor Center
Sun Yat-Sen Memorial Hospital

Jiang Liu

Reply | Reply All | Forward | Move | Mark | More | Delete

回复:

2022-11-02 19:35:59

From : "刘江" <zyliujiang@163.com>

To : "黄迪" <huangd63@mail.sysu.edu.cn>

Agree! Thank you!

Jiang Liu
Breast Tumor Center&Erwei Song Lab,Sun Yat-Sen Memorial Hospital,
Sun Yat-Sen University, Guangzhou 510120, China
Phone: (+86)20-87332022.

在 2022-11-02 15:15:54，"黄迪" <huangd63@mail.sysu.edu.cn> 写道：

> Dear Dr Liu,
>
> We are planning to submit our manuscript entitled "CCL18 signaling from tumor-associated macrophages activates fibroblasts to adopt a chemoresistance-inducing phenotype" back to *Oncogene*. And our final author list is as below：
>
> Wenfeng Zeng, Lixiong Xiong, Wei Wu, Shunrong Li, Jiang Liu, Linbing Yang, Liyan Lao, Mengmeng Zhang, Penghan Huang, Huiping Chen, Nanyan Miao, Zifei Liu, Xinyu Yang, Zhirong Lin, Jiayi Wang, Pei Wang, Erwei Song, Yan-Dan Yao，Yan Nie，Jianing Chen and Di huang
>
> Please reply to my email confirming that you agree to these changes of the author list. And please feel free to contact us with any questions.
>
> Look forward to hearing from you soon.
>
> Best wishes.
>
> Di Huang
>
> Di Huang, PhD
> Breast Tumor Center
> Sun Yat-Sen Memorial Hospital

Linbing Yang

Reply | Reply All | Forward | Move | Mark | More | Delete

回复：(无主题)

2022-11-02 15:39:24

From : "杨林槟" <yanglinb123@163.com>

To : "黄迪" <huangd63@mail.sysu.edu.cn>

I agree

---- 回复的原邮件 ----

发件人 黄迪<huangd63@mail.sysu.edu.cn>

发送日期 2022年11月02日 15:17

收件人 yanglinb123<yanglinb123@163.com>

主题

Dear Dr Yang,

We are planning to submit our manuscript entitled "CCL18 signaling from tumor-associated macrophages activates fibroblasts to adopt a chemoresistance-inducing phenotype" back to *Oncogene*. And our final author list is as below：

Wenfeng Zeng, Lixiong Xiong, Wei Wu, Shunrong Li, Jiang Liu, Linbing Yang, Liyan Lao, Mengmeng Zhang, Penghan Huang, Huiping Chen, Nanyan Miao, Zifei Liu, Xinyu Yang, Zhirong Lin, Jiayi Wang, Pei Wang, Erwei Song, Yan-Dan Yao， Yan Nie， Jianing Chen and Di huang

Please reply to my email confirming that you agree to these changes of the author list. And please feel free to contact us with any questions.

Look forward to hearing from you soon.

Best wishes.

Di Huang

Di Huang, PhD
Breast Tumor Center
Sun Yat-Sen Memorial Hospital

Liyan Lao

Reply | Reply All | Forward | Move | Mark | More | Delete

回复:(无主题)

2022-11-02 16:09:20

From : "劳丽燕" <laoly@mail2.sysu.edu.cn>

To : "黄迪" <huangd63@mail.sysu.edu.cn>

Agree

--------------原始邮件--------------
发件人："黄迪 "<huangd63@mail.sysu.edu.cn>;
发送时间：2022年11月2日(星期三) 下午3:18
收件人："劳丽燕" <laoly@mail2.sysu.edu.cn>;
主题：

Dear Dr Lao,

We are planning to submit our manuscript entitled "CCL18 signaling from tumor-associated macrophages activates fibroblasts to adopt a chemoresistance-inducing phenotype" back to *Oncogene*. And our final author list is as below:

Wenfeng Zeng, Lixiong Xiong, Wei Wu, Shunrong Li, Jiang Liu, Linbing Yang, Liyan Lao, Mengmeng Zhang, Penghan Huang, Huiping Chen, Nanyan Miao, Zifei Liu, Xinyu Yang, Zhirong Lin, Jiayi Wang, Pei Wang, Erwei Song, Yan-Dan Yao, Yan Nie, Jianing Chen and Di huang

Please reply to my email confirming that you agree to these changes of the author list. And please feel free to contact us with any questions.

Look forward to hearing from you soon.

Best wishes.

Di Huang

Di Huang, PhD
Breast Tumor Center
Sun Yat-Sen Memorial Hospital

Mengmeng Zhang

Reply | Reply All | Forward | Move | Mark | More | Delete

Re:

2022-11-02 17:59:41

From : "张萌萌" <zhang_mengmeng@pku.edu.cn>

To : "黄迪" <huangd63@mail.sysu.edu.cn>

Agree

-----原始邮件-----
发件人:"黄迪" <huangd63@mail.sysu.edu.cn>
发送时间:2022-11-02 15:19:01 (星期三)
收件人: zhang_mengmeng@pku.edu.cn
抄送:
主题:

Dear Dr Zhang,

We are planning to submit our manuscript entitled "CCL18 signaling from tumor-associated macrophages activates fibroblasts to adopt a chemoresistance-inducing phenotype" back to *Oncogene*. And our final author list is as below:

Wenfeng Zeng, Lixiong Xiong, Wei Wu, Shunrong Li, Jiang Liu, Linbing Yang, Liyan Lao, Mengmeng Zhang, Penghan Huang, Huiping Chen, Nanyan Miao, Zifei Liu, Xinyu Yang, Zhirong Lin, Jiayi Wang, Pei Wang, Erwei Song, Yan-Dan Yao, Yan Nie, Jianing Chen and Di huang

Please reply to my email confirming that you agree to these changes of the author list. And please feel free to contact us with any questions.

Look forward to hearing from you soon.

Best wishes.

Di Huang

Di Huang, PhD
Breast Tumor Center
Sun Yat-Sen Memorial Hospital

Penghan Huang

Reply | Reply All | Forward | Move | Mark | More | Delete

回复:(无主题)

2022-11-02 15:30:57

From : "黄鹏翰" <huangph5@mail2.sysu.edu.cn>

To : "黄迪" <huangd63@mail.sysu.edu.cn>

Dear Dr. Huang,

I agree the author list.

Best wishes.

Penghan Huang

--------------原始邮件--------------
发件人："黄迪 "<huangd63@mail.sysu.edu.cn>;
发送时间：2022年11月2日(星期三) 下午3:16
收件人："黄鹏翰" <huangph5@mail2.sysu.edu.cn>;
主题：

Dear Mr Huang,

We are planning to submit our manuscript entitled "CCL18 signaling from tumor-associated macrophages activates fibroblasts to adopt a chemoresistance-inducing phenotype" back to *Oncogene*. And our final author list is as below:

Wenfeng Zeng, Lixiong Xiong, Wei Wu, Shunrong Li, Jiang Liu, Linbing Yang, Liyan Lao, Mengmeng Zhang, Penghan Huang, Huiping Chen, Nanyan Miao, Zifei Liu, Xinyu Yang, Zhirong Lin, Jiayi Wang, Pei Wang, Erwei Song, Yan-Dan Yao, Yan Nie, Jianing Chen and Di huang

Please reply to my email confirming that you agree to these changes of the author list. And please feel free to contact us with any questions.

Look forward to hearing from you soon.

Best wishes.

Di Huang

Di Huang, PhD
Breast Tumor Center
Sun Yat-Sen Memorial Hospital

Huiping Chen

Reply | Reply All | Forward | Move | Mark | More | Delete

Re:

2022-11-02 15:24:51

From : "陈惠萍" <chenhp8@mail2.sysu.edu.cn>

To : "黄迪" <huangd63@mail.sysu.edu.cn>

Yes, I agree.

发自我的iPhone

------------------ Original ------------------

From: 黄迪 <huangd63@mail.sysu.edu.cn>
Date: Wed,Nov 2,2022 3:20 PM
To: chenhp8 <chenhp8@mail2.sysu.edu.cn>
Subject: Re:

Dear Dr Chen,

We are planning to submit our manuscript entitled "CCL18 signaling from tumor-associated macrophages activates fibroblasts to adopt a chemoresistance-inducing phenotype" back to Oncogene. And our final author list is as below:

Wenfeng Zeng, Lixiong Xiong, Wei Wu, Shunrong Li, Jiang Liu, Linbing Yang, Liyan Lao, Mengmeng Zhang, Penghan Huang, Huiping Chen, Nanyan Miao, Zifei Liu, Xinyu Yang, Zhirong Lin, Jiayi Wang, Pei Wang, Erwei Song, Yan-Dan Yao, Yan Nie, Jianing Chen and Di huang

Please reply to my email confirming that you agree to these changes of the author list. And please feel free to contact us with any questions.

Look forward to hearing from you soon.

Best wishes.

Di Huang

Di Huang, PhD
Breast Tumor Center
Sun Yat-Sen Memorial Hospital

Nanyan Miao

Reply | Reply All | Forward | Move | Mark | More | Delete

回复:(无主题)

2022-11-02 15:42:39

From : "缪楠焱" <miaony@mail2.sysu.edu.cn>

To : "黄迪" <huangd63@mail.sysu.edu.cn>

agree

--------------原始邮件--------------
发件人："黄迪 "<huangd63@mail.sysu.edu.cn>;
发送时间：2022年11月2日(星期三) 下午3:20
收件人："缪楠焱"<miaony@mail2.sysu.edu.cn>;
主题：

Dear Dr Miao,

We are planning to submit our manuscript entitled "CCL18 signaling from tumor-associated macrophages activates fibroblasts to adopt a chemoresistance-inducing phenotype" back to *Oncogene*. And our final author list is as below:

Wenfeng Zeng, Lixiong Xiong, Wei Wu, Shunrong Li, Jiang Liu, Linbing Yang, Liyan Lao, Mengmeng Zhang, Penghan Huang, Huiping Chen, Nanyan Miao, Zifei Liu, Xinyu Yang, Zhirong Lin, Jiayi Wang, Pei Wang, Erwei Song, Yan-Dan Yao, Yan Nie, Jianing Chen and Di huang

Please reply to my email confirming that you agree to these changes of the author list. And please feel free to contact us with any questions.

Look forward to hearing from you soon.

Best wishes.

Di Huang

Di Huang, PhD
Breast Tumor Center
Sun Yat-Sen Memorial Hospital

Zifei Liu

Reply | Reply All | Forward | Move | Mark | More | Delete

Re:

刘子菲 send to 黄迪

2022-11-02 19:40:29

agree

------------------ Original ------------------

From: "黄迪"<huangd63@mail.sysu.edu.cn>;
Date: Wed, Nov 2, 2022 03:21 PM
To: "刘子菲"<liuzf5@mail2.sysu.edu.cn>;
Subject:

Dear Miss Liu,

We are planning to submit our manuscript entitled "CCL18 signaling from tumor-associated macrophages activates fibroblasts to adopt a chemoresistance-inducing phenotype" back to *Oncogene*. And our final author list is as below:

Wenfeng Zeng, Lixiong Xiong, Wei Wu, Shunrong Li, Jiang Liu, Linbing Yang, Liyan Lao, Mengmeng Zhang, Penghan Huang, Huiping Chen, Nanyan Miao, Zifei Liu, Xinyu Yang, Zhirong Lin, Jiayi Wang, Pei Wang, Erwei Song, Yan-Dan Yao, Yan Nie, Jianing Chen and Di huang

Please reply to my email confirming that you agree to these changes of the author list. And please feel free to contact us with any questions.

Look forward to hearing from you soon.

Best wishes.

Di Huang

Di Huang, PhD
Breast Tumor Center
Sun Yat-Sen Memorial Hospital

Xinyu Yang

Reply | Reply All | Forward | Move | Mark | More | Delete

回复:(无主题)

2022-11-02 15:26:35

From : "杨欣瑜" <yangxy73@mail2.sysu.edu.cn>

To : "黄迪" <huangd63@mail.sysu.edu.cn>

agree

--------------原始邮件--------------
发件人："黄迪 "<huangd63@mail.sysu.edu.cn>;
发送时间：2022年11月2日(星期三) 下午3:24
收件人："杨欣瑜"<yangxy73@mail2.sysu.edu.cn>;
主题：

Dear Miss Yang,

We are planning to submit our manuscript entitled "CCL18 signaling from tumor-associated macrophages activates fibroblasts to adopt a chemoresistance-inducing phenotype" back to *Oncogene*. And our final author list is as below：

Wenfeng Zeng, Lixiong Xiong, Wei Wu, Shunrong Li, Jiang Liu, Linbing Yang, Liyan Lao, Mengmeng Zhang, Penghan Huang, Huiping Chen, Nanyan Miao, Zifei Liu, Xinyu Yang, Zhirong Lin, Jiayi Wang, Pei Wang, Erwei Song, Yan-Dan Yao， Yan Nie，Jianing Chen and Di huang

Please reply to my email confirming that you agree to these changes of the author list. And please feel free to contact us with any questions.

Look forward to hearing from you soon.

Best wishes.

Di Huang

Di Huang, PhD
Breast Tumor Center
Sun Yat-Sen Memorial Hospital

Zhirong Lin

Reply | Reply All | Forward | Move | Mark | More | Delete

回复:

2022-11-02 15:51:50

From : "林治荣" <linzhr3@mail2.sysu.edu.cn>

To : "黄迪" <huangd63@mail.sysu.edu.cn>

agree

----原始邮件----

发件人: "黄迪"<huangd63@mail.sysu.edu.cn>
发送时间: 2022年11月2日(周三) 下午3:25
收件人: "linzhr3"<linzhr3@mail2.sysu.edu.cn>;
主题: null

Dear Dr Lin,

We are planning to submit our manuscript entitled "CCL18 signaling from tumor-associated macrophages activates fibroblasts to adopt a chemoresistance-inducing phenotype" back to Oncogene. And our final author list is as below:

Wenfeng Zeng, Lixiong Xiong, Wei Wu, Shunrong Li, Jiang Liu, Linbing Yang, Liyan Lao, Mengmeng Zhang, Penghan Huang, Huiping Chen, Nanyan Miao, Zifei Liu, Xinyu Yang, Zhirong Lin, Jiayi Wang, Pei Wang, Erwei Song, Yan-Dan Yao, Yan Nie, Jianing Chen and Di huang

Please reply to my email confirming that you agree to these changes of the author list. And please feel free to contact us with any questions.

Look forward to hearing from you soon.

Best wishes.

Di Huang

Di Huang, PhD
Breast Tumor Center
Sun Yat-Sen Memorial Hospital

Jiayi Wang

Reply | Reply All | Forward | Move | Mark | More | Delete

回复：无主题

2022-11-02 19:34:50

From : "王家宜" <mn3266@163.com>

To : "黄迪" <huangd63@mail.sysu.edu.cn>

agree

---- 回复的原邮件 ----

发件人	黄迪<huangd63@mail.sysu.edu.cn>
日期	2022年11月02日 15:26
收件人	mn3266@163.com<mn3266@163.com>
主题	（无主题）

Dear Miss Wang,

We are planning to submit our manuscript entitled "CCL18 signaling from tumor-associated macrophages activates fibroblasts to adopt a chemoresistance-inducing phenotype" back to *Oncogene*. And our final author list is as below：

Wenfeng Zeng, Lixiong Xiong, Wei Wu, Shunrong Li, Jiang Liu, Linbing Yang, Liyan Lao, Mengmeng Zhang, Penghan Huang, Huiping Chen, Nanyan Miao, Zifei Liu, Xinyu Yang, Zhirong Lin, Jiayi Wang, Pei Wang, Erwei Song, Yan-Dan Yao， Yan Nie，Jianing Chen and Di huang

Please reply to my email confirming that you agree to these changes of the author list. And please feel free to contact us with any questions.

Look forward to hearing from you soon.

Best wishes.

Di Huang

Di Huang, PhD
Breast Tumor Center
Sun Yat-Sen Memorial Hospital

Pei Wang

Reply | Reply All | Forward | Move | Mark | More | Delete

Re:

2022-11-02 15:28:34

From : "wangpei20210512" <wangpei20210512@163.com>

To : "黄迪" <huangd63@mail.sysu.edu.cn>

agree

在 2022-11-02 15:27:41，"黄迪" <huangd63@mail.sysu.edu.cn> 写道：

> Dear Miss Wang pei,
>
> We are planning to submit our manuscript entitled "CCL18 signaling from tumor-associated macrophages activates fibroblasts to adopt a chemoresistance-inducing phenotype" back to *Oncogene*. And our final author list is as below：
>
> Wenfeng Zeng, Lixiong Xiong, Wei Wu, Shunrong Li, Jiang Liu, Linbing Yang, Liyan Lao, Mengmeng Zhang, Penghan Huang, Huiping Chen, Nanyan Miao, Zifei Liu, Xinyu Yang, Zhirong Lin, Jiayi Wang, Pei Wang, Erwei Song, Yan-Dan Yao，Yan Nie，Jianing Chen and Di huang
>
> Please reply to my email confirming that you agree to these changes of the author list. And please feel free to contact us with any questions.
>
> Look forward to hearing from you soon.
>
> Best wishes.
>
> Di Huang
>
> Di Huang, PhD
> Breast Tumor Center
> Sun Yat-Sen Memorial Hospital

Erwei Song

Reply | Reply All | Forward | Move | Mark | More | Delete

Re:

2022-11-02 14:18:13

From : "宋尔卫" <songew@mail.sysu.edu.cn>

To : "黄迪" <huangd63@mail.sysu.edu.cn>

Agree.

Erwei Song

Professor of Breast Surgery
Sun Yat-sen Memorial Hospital,
Sun Yat-sen University
107 Yanjiang West Rd
Guangzhou, 510120
China
e-mail: songew@mail.sysu.edu.cn

-----原始邮件-----
发件人:"黄迪" <huangd63@mail.sysu.edu.cn>
发送时间:2022-11-02 13:25:08 (星期三)
收件人: songew@mail.sysu.edu.cn
抄送:
主题:

Dear professor Song,

We are planning to submit our manuscript entitled "CCL18 signaling from tumor-associated macrophages activates fibroblasts to adopt a chemoresistance-inducing phenotype" back to *Oncogene*. And our final author list is as below:

Wenfeng Zeng, Lixiong Xiong, Wei Wu, Shunrong Li, Jiang Liu, Linbing Yang, Liyan Lao, Mengmeng Zhang, Penghan Huang, Huiping Chen, Nanyan Miao, Zifei Liu, Xinyu Yang, Zhirong Lin, Jiayi Wang, Pei Wang, Erwei Song, Yan-Dan Yao, Yan Nie, Jianing Chen and Di huang

Please reply to my email confirming that you agree to these changes of the author list. And please feel free to contact us with any questions.

Look forward to hearing from you soon.

Best wishes.

Di Huang

Di Huang, PhD
Breast Tumor Center
Sun Yat-Sen Memorial Hospital

Yandan Yao

Reply | Reply All | Forward | Move | Mark | More | Delete

Re:

2022-11-02 15:32:36

From : "Yandan Yao" <yaoyand@mail.sysu.edu.cn>

To : "黄迪" <huangd63@mail.sysu.edu.cn>

I agree.

-----原始邮件-----
发件人:"黄迪" <huangd63@mail.sysu.edu.cn>
发送时间:2022-11-02 15:29:52 (星期三)
收件人: yaoyand@mail.sysu.edu.cn
抄送:
主题:

Dear professor Yao,

We are planning to submit our manuscript entitled "CCL18 signaling from tumor-associated macrophages activates fibroblasts to adopt a chemoresistance-inducing phenotype" back to *Oncogene*. And our final author list is as below:

Wenfeng Zeng, Lixiong Xiong, Wei Wu, Shunrong Li, Jiang Liu, Linbing Yang, Liyan Lao, Mengmeng Zhang, Penghan Huang, Huiping Chen, Nanyan Miao, Zifei Liu, Xinyu Yang, Zhirong Lin, Jiayi Wang, Pei Wang, Erwei Song, Yan-Dan Yao, Yan Nie, Jianing Chen and Di huang

Please reply to my email confirming that you agree to these changes of the author list. And please feel free to contact us with any questions.

Look forward to hearing from you soon.

Best wishes.

Di Huang

Di Huang, PhD
Breast Tumor Center
Sun Yat-Sen Memorial Hospital

Yan Nie

Reply | Reply All | Forward | Move | Mark | More | Delete

回复： （无主题）

2022-11-02 15:39:20

From : "nieyan7" <nieyan7@mail.sysu.edu.cn>

To : "黄迪" <huangd63@mail.sysu.edu.cn>

Dear Prof. Huang

I agree to the changes of the author list.
Thank you!

Beat regards
Yan Nie

---- 回复的原邮件 ----

发件人	黄迪<huangd63@mail.sysu.edu.cn>
发送日期	2022年11月2日 15:30
收件人	<nieyan7@mail.sysu.edu.cn>
主题	(无主题)

Dear professor Nie,

We are planning to submit our manuscript entitled "CCL18 signaling from tumor-associated macrophages activates fibroblasts to adopt a chemoresistance-inducing phenotype" back to *Oncogene*. And our final author list is as below:

Wenfeng Zeng, Lixiong Xiong, Wei Wu, Shunrong Li, Jiang Liu, Linbing Yang, Liyan Lao, Mengmeng Zhang, Penghan Huang, Huiping Chen, Nanyan Miao, Zifei Liu, Xinyu Yang, Zhirong Lin, Jiayi Wang, Pei Wang, Erwei Song, Yan-Dan Yao, Yan Nie, Jianing Chen and Di huang

Please reply to my email confirming that you agree to these changes of the author list. And please feel free to contact us with any questions.

Look forward to hearing from you soon.

Best wishes.

Di Huang

Di Huang, PhD
Breast Tumor Center
Sun Yat-Sen Memorial Hospital

Jianing Chen

Reply | Reply All | Forward | Move | Mark | More | Delete

Re:

2022-11-02 15:12:44

From : "陈嘉宁" <chenjn335@mail.sysu.edu.cn>

To : "黄迪" <huangd63@mail.sysu.edu.cn>

agree

-----原始邮件-----
发件人:"黄迪" <huangd63@mail.sysu.edu.cn>
发送时间:2022-11-02 15:10:33 (星期三)
收件人: chenjn335@mail.sysu.edu.cn
抄送:
主题:

Dear Ms Chen,

We are planning to submit our manuscript entitled "CCL18 signaling from tumor-associated macrophages activates fibroblasts to adopt a chemoresistance-inducing phenotype" back to *Oncogene*. And our final author list is as below:

Wenfeng Zeng, Lixiong Xiong, Wei Wu, Shunrong Li, Jiang Liu, Linbing Yang, Liyan Lao, Mengmeng Zhang, Penghan Huang, Huiping Chen, Nanyan Miao, Zifei Liu, Xinyu Yang, Zhirong Lin, Jiayi Wang, Pei Wang, Erwei Song, Yan-Dan Yao, Yan Nie, Jianing Chen and Di huang

Please reply to my email confirming that you agree to these changes of the author list. And please feel free to contact us with any questions.

Look forward to hearing from you soon.

Best wishes.

Di Huang

Di Huang, PhD
Breast Tumor Center
Sun Yat-Sen Memorial Hospital
